# CXCL10^high^TNFα^high^Ki67 ^+^ Microglia Recruit and Activate CD8 ^+^ T Cells in the Brainstem During Experimental Cerebral Malaria

**DOI:** 10.1111/cns.70425

**Published:** 2025-06-02

**Authors:** Yi Wang, Jiao Liang, Chao Yang, Jun Wang, Qinghao Zhu, Guodong Tong, Tong Li, Ganze Li, Yuxiao Huang, Yimin Yang, Jie Ren, Yinghui Li, Yan Shen, Ya Zhao

**Affiliations:** ^1^ Department of Medical Microbiology and Parasitology Air Force Medical University Xi'an Shaanxi China; ^2^ College of Life Sciences, Northwest University Xi'an Shaanxi China; ^3^ College of Basic Medical Sciences, Yan'an University Yan'an Shaanxi China; ^4^ Grade 2022 Clinical Medicine (Five‐Year Program), basic Medical College, Air Force Medical University Xi'an Shaanxi China; ^5^ Health Service Training Base Air Force Medical University Xi'an Shaanxi China

**Keywords:** activation, CD8^+^ T cell, experimental cerebral malaria, heterogeneity, microglia, recruitment

## Abstract

**Aims:**

This study aimed to investigate the heterogeneity of microglia and their role in recruiting and activating CD8^+^ T cells in experimental cerebral malaria (ECM).

**Methods:**

C57BL/6J mice were infected with *Plasmodium berghei* ANKA (PbA) to induce ECM. Morphology and distribution of microglia were assessed via immunofluorescence (IF) staining and electron microscopy. Single‐cell RNA‐sequencing (scRNA‐seq) analyzed the activation characteristics of infiltrating CD8^+^ T cells and the transcriptional heterogeneity of microglia in ECM. In vitro, ECM‐associated microglia were induced by TNFα, IFNγ, and parasite‐infected red blood cells (pRBCs). The interaction between microglia and CD8^+^ T cells was explored in co‐culture systems through transwell assay, adhesion assay, and cytotoxicity test.

**Results:**

In vivo, microglia were aggregated in the brainstem and olfactory bulb in the ECM brain, regions that exhibited more severe pathological injury. The transcriptional characteristics of ECM microglia distinguished from physiological microglia and exhibit morphological heterogeneity in the ECM brain. Infiltrating CD8^+^ T cells in the ECM brainstem exhibit sustained activation characteristics associated with microglia interaction. Based on subcluster analysis, a unique subtype of ECM‐associated microglia was identified, characterized by CXCL10^high^TNFα^high^Ki67^+^. These microglia mediated the recruitment and sustained activation of CD8^+^ T cells through persistent interactions in co‐culture systems.

**Conclusions:**

Our study identified an ECM‐associated microglia subtype and explored its interaction with CD8^+^ T cells, which deepened the understanding of the multifaceted role of microglia in the pathogenesis of CM neuroinflammation.

## Graphical abstract

Microglia exhibit distinct transcriptional and morphological heterogeneity in experimental cerebral malaria (ECM) compared to physiological microglia. A unique subtype of ECM‐associated microglia, characterized by CXCL10^high^TNFα^high^Ki67^+^, mediates the recruitment and sustained activation of CD8^+^ T cells through ongoing interactions, exacerbating neuropathology.

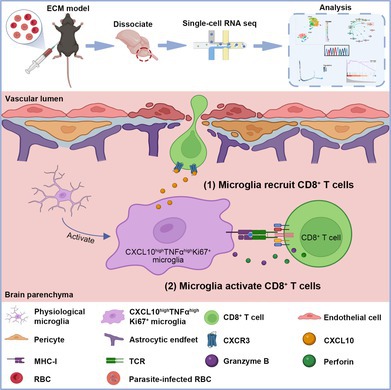

## 1 Introduction

Malaria is a mosquito‐borne disease caused by protozoan parasites of the genus *Plasmodium*. According to the latest report of WHO, it caused 249 million cases and 608,000 deaths worldwide in 2022 [1]. Cerebral malaria (CM) is an infrequent but life‐threatening complication of severe malaria that presents as an acute cerebrovascular encephalopathy characterized by unarousable coma [1, 2]. Despite effective antimalarial drug treatment, 20% of patients with CM die from this disease, and many survivors have neurocognitive impairment [3]. Current studies suggest that the primary pathogenesis of CM is the sequestration of parasite‐infected red blood cells (pRBCs) and immune cells on brain endothelial cells, which synergistically leads to the disruption of the blood–brain barrier (BBB) and potentially neuropathology [4]. There is growing evidence that the ECM‐activated CD8^+^ T cell‐mediated cytotoxic effect plays a crucial role in CM progression [5, 6, 7], in stark contrast to other neuroinflammation and neurodegeneration diseases, where CD4^+^ T cells are predominantly mobilized within the brain [8, 9, 10]. Our previous research also confirmed that T lymphocytes recruited to the brains of experimental cerebral malaria (ECM) mice were almost CD8^+^ T cells, rather than CD4^+^ T cells [11]. This distinction highlights the unique immunological landscape and complexity of CM. However, the precise mechanisms underlying the recruitment of ECM‐activated CD8^+^ T cells into parenchyma and their sustained activation within the brain remain poorly understood.

Microglia, the tissue‐resident macrophages in the CNS [12, 13, 14, 15], are essential for CNS homeostasis [16]. Serving as the initial line of defense against insults to the brain, the activation of microglia is intricately linked to the progression of neurodegenerative and neuroinflammatory diseases [17]. Microglia exhibit distinct phenotypes, functions, and transcriptomic states, particularly during developmental stages [18, 19], aging [20], and under varying pathological conditions [21, 22]. However, due to the complexity and specificity of the immune microenvironment in CNS diseases, classic macrophage classification methods based on cell‐surface markers (“resting,” “M1,” or “M2”) are no longer able to accurately characterize the heterogeneity of microglia and the complexity of their functional states [23]. Single‐cell RNA‐sequencing (scRNA‐seq) technology offers significant advantages in studying cellular heterogeneity and expression differences [24, 25], enabling the identification of cell types and subtypes based on their unique transcriptional features. For instance, in the context of Alzheimer's disease (AD), scRNA‐seq analysis has revealed a novel disease‐associated microglia type, which has the potential to restrict neurodegeneration [26]. Similarly, diverse microglia subtypes have been identified in both healthy and multiple sclerosis brain [18, 27]. Although scRNA‐seq analysis of microglia has been conducted across various disease models, the defining characteristics of microglia subtypes, their specific activation patterns, and their intricate interplay with the pathological manifestations associated with CD8^+^ T cell recruitment remain largely elusive in ECM.

In ECM mice, ECM‐activated CD8^+^ T cells are attracted by chemokines released from activated endothelial cells and subsequently adhere to and interact with the brain endothelium, resulting in endothelial injury and disintegration of the BBB [28, 29, 30]. Of note, a considerable number of CD8^+^ T cells were found to infiltrate the brain parenchyma in both CM death patients and ECM mice [6, 31, 32]. Our prior investigations into ECM have established that these infiltrating CD8^+^ T cells are proliferative and secrete effector molecules like IFNγ, leading to neuronal death, implying a mechanism in the brain that perpetuates the activation of CD8^+^ T cells [11, 33, 34]. As the main antigen‐presenting cells in the brain, microglia play a pivotal role in the recruitment and activation of T cells [35]. In AD, activated microglia recruit T cells, while CD8^+^ T cells amplify inflammatory signaling and antigen‐presentation capabilities of microglia through IFNγ secretion [36]. In a nasal virus‐infected mouse model, microglia were found to cross‐present antigen to virus‐specific CD8^+^ T cells after acquiring antigen from adjacent neurons [37]. Through morphology, transcriptome analysis, and experimental evidence, the role of microglia in the development of ECM neuroinflammation has been preliminarily characterized [38, 39, 40, 41]. However, the role of microglia in the recruitment and maintenance activation of infiltrating CD8^+^ T cells in ECM has not been reported.

In this study, based on the ECM mouse model and scRNA‐seq analysis, we sought to elucidate the transcriptional signatures of microglia and their interaction with CD8^+^ T cells in the brainstem of ECM mice. Our findings revealed a distinct subtype of ECM‐associated microglia characterized by high expression of CXCL10, TNFα, and Ki67. This subtype exhibited proliferative and pro‐inflammatory properties and was intimately linked to the recruitment of CD8^+^ T cells in the brain parenchyma. Further in vitro experiments corroborated that these microglia were capable of recruiting and sustaining the activation of ECM‐activated CD8^+^ T cells. However, this process was accompanied by damage to the microglia themselves, further exacerbating neuropathological conditions. Overall, we defined the molecular characteristics of microglia in the ECM neuroinflammatory microenvironment using scRNA‐seq and first identified a specific type of microglia associated with CD8^+^ T cell‐mediated ECM immunopathology. Our results provide a new theoretical basis for further revealing the potential role of microglia in the immunopathogenesis of CM and provide new insights into immunotherapy for CM neurological symptoms.

## 2 Materials and Methods

### 2.1 Ethics Statement

The Institutional Review Board of the Air Force Medical University (No. IACUC‐20200407) granted approval for the animal experiments conducted in this study. All procedures were implemented with the aim of minimizing harm to the experimental mice.

### 2.2 Mice and Infection

C57BL/6J male mice were procured from the Experimental Animal Center of the Air Force Medical University. B6.129P‐Cx3cr1^*tm1Litt*^/J transgenic (CX3CR1‐GFP) mice were provided by Dr. Hongyan Qin (Department of Medical Genetics and Developmental Biology, Air Force Medical University, Xi'an, China). The mice were fed in a specific pathogen‐free system, providing adequate water and food, and were randomly numbered and grouped. The *Plasmodium berghei* ANKA (PbA) strain was preserved in liquid nitrogen, maintained, and used according to previously established protocols [41]. Male mice, aged 5–6 weeks and weighing between 18 and 20 g, were injected intraperitoneally (i.p.) with 5 × 10^6^ pRBCs to induce ECM. The control group did not receive any treatment, and all groups were provided with standard feeding. On the 7th day post‐infection, the mice were euthanized, and their entire brains were collected for subsequent experimental analysis following cardiac perfusion with normal saline.

### 2.3 Antibodies and Reagents

The primary antibodies against mouse CD8 (GB13429), Ki67 (GB111141), and TNFα (GB12188) were purchased from ServiceBio (China). Antibodies against mouse CD18 (10554‐1‐AP) and CXCL10 (10937‐1‐AP) were purchased from Proteintech (China). Antibodies against HLA Class 1 ABC (EM1801‐10) and mouse IFNγ (A12450) were purchased from HUABIO (China) and ABclonal (China), respectively. Fluorescence‐labeled secondary antibodies, including FITC‐conjugated goat anti‐mouse IgG (GB22301), FITC‐conjugated goat anti‐rabbit IgG (GB22303), Cy3‐conjugated goat anti‐mouse IgG (GB21301), and Cy3‐conjugated goat anti‐rabbit IgG (GB21303) for immunofluorescence staining, were purchased from ServiceBio (China). PE‐labeled antibody against mouse CD8 (100707), FITC‐labeled CD44 (163605), and APC‐labeled CD69 (104513) for flow cytometry (FC) were purchased from BioLegend (USA). IFNγ (50709‐MNAH) was purchased from SinoBiological (China) and TNFα (05–168) was from Sigma‐Aldrich (USA).

### 2.4 Transmission Electron Microscopy

Mice were anesthetized and sacrificed on the 7th day post‐infection, and their brainstems were promptly isolated. The brainstems were fixed in 2.5% glutaraldehyde (P1126, Solarbio, China) at 4°C for 2 h, and subsequently washed multiple times with PBS post‐fixation. The tissues underwent a progressive dehydration process using 50%, 75%, 95%, and 100% ethanol, were embedded in epoxy resin, and then sectioned into 0.5‐μm slices utilizing an ultra‐microtome for electron microscopy (JEM‐1400PLUS, JEOL, Japan).

### 2.5 Hematoxylin and Eosin (H&E) Staining

Mice were perfused with normal saline via the heart, and their entire brains were fixed in 4% paraformaldehyde (PFA) overnight. Following dehydration, transparency treatment, and paraffin embedding, the brain tissues were sectioned into 5‐μm slices and subsequently mounted on slides. After undergoing deparaffinization and rehydration, these sections were utilized for H&E staining. Sections were stained with hematoxylin for 10–15 min, differentiated with 1% hydrochloric acid alcohol for a few seconds, and returned blue with 0.6% aqueous ammonia. Rinse the sections with running water after each step. Then, sections were stained with eosin for 1–3 min. After dehydration and transparency, sections were sealed with neutral resins.

### 2.6 Immunofluorescent (IF) Staining

Paraffin‐embedded sections of mouse brain tissue were prepared according to the previously described methodology. Microglia were seeded and cultured on coverslips within 24‐well plates that had been coated with poly‐D‐lysine (A38904‐01, Gibco, USA). After various cell treatments, the coverslips were fixed in 4% PFA for 20 min. Samples on the coverslips and the sections on the slides underwent sequential incubation in a blocking buffer containing 2% bovine serum, 3% bovine serum albumin, and 0.2% Triton X‐100 for 1 h, followed by incubation with primary antibodies overnight and secondary antibodies for 2 h. All antibodies were diluted in the aforementioned blocking buffer. Subsequently, the coverslips and sections were mounted in a mounting medium containing DAPI (ab104139, Abcam, UK). Digital slide scanning and image acquisition were performed using the Pannoramic DESK (P‐MIDI, P25, Japan) and CaseViewer 2.4 (3DHISTECH, Hungary), respectively. The acquired images were analyzed using ImageJ 1.53c.

### 2.7 Primary Cortical Microglia Isolation and Culture

Mouse brains were aseptically collected within 3 days postnatally and placed in cold D‐Hank's balanced salt solution. The cerebral cortices were isolated microscopically, with meninges and blood vessels removed as thoroughly as possible. Single cells were mechanically separated by the repeated blowing and sucking of the tissue block using 1‐mL pipette tips, centrifuged, and resuspended in Dulbecco's modified Eagle's medium (DMEM) (H30284.01, Hyclone, China) with 10% fetal bovine serum (FBS) (F103‐01, Vazyme, China), 2 mM L‐glutamine (25030–164, Gibco, USA), and 1% penicillin and streptomycin (G4003, ServiceBio, China). Cells collected from 3 to 4 mice were seeded on one poly‐D‐lysine coated 75 cm^2^ cell culture flask and cultured in a 5% CO_2_ incubator at 37°C. After 2 weeks, the cells grew in layers, with adherent astrocytes at the bottom and bright round microglia at the top, with medium renewed every 3 days. Microglia were isolated and obtained by shaking the culture flask at 200 rpm for 2 h, collecting supernatants and centrifuging.

### 2.8 Purification of Spleen CD8 ^+^ T Cells

Mice infected with PbA for 5–8 days were anesthetized and subsequently sacrificed to isolate intact spleens. The spleens were homogenized in cold PBS and filtered through a 200‐mesh metal sieve to create a tissue suspension. This suspension was then subjected to centrifugation, and the resulting precipitate was resuspended in erythrocyte lysis buffer (AR1118, Bosterbio, China). CD8^+^ T cells were isolated from the suspension using a magnetic bead sorting kit (558471, BD, USA) with negative selection.

### 2.9 Cells Co‐Culture and Transwell Co‐Culture System

In the co‐culture system, microglia were co‐stimulated with TNFα (10 ng/mL), IFNγ (10 ng/mL), and pRBC (pRBC: microglia = 20: 1) for 24 h, and then CD8^+^ T cells were added at an effector‐to‐target cell ratio of 10:1. Total RNA and supernatants were separated and collected for subsequent tests after co‐culture. CD8^+^ T cells used for cell adhesion experiments were isolated from wild‐type or GFP transgenic mice. In the Transwell system, microglia were seeded on a 6‐well plate, with or without co‐stimulation of TNFα, IFNγ, and pRBC for 24 h, and CD8^+^ T cells were then cultured in the Transwell chamber with a 5.0‐μm aperture for 4 h.

### 2.10 Quantitative Real‐Time PCR (q‐PCR)

Total RNA was extracted utilizing the TRIZOL reagent method and subsequently reverse transcribed into cDNA using reverse transcription supermix (R222‐01, Vazyme, China). q‐PCR was performed using a 2 × SYBR q‐PCR master mix (Q311‐02, Vazyme, China). The primers, detailed in Table S1, were synthesized by Sangon Biotech (China). Relative gene expression was quantified using the 2^−ΔΔCT^ method, with β‐actin serving as the endogenous reference.

### 2.11 Flow Cytometry (FC)

Treat cells according to experimental requirements. The CD8^+^ T cells in supernatant were collected by centrifugation. Adherent cells were digested with 0.25% trypsin, centrifuged, and resuspended in D‐Hank's solution with 0.5% FBS. Cells were incubated with the above antibodies for 30 min and then resuspended in D‐Hank's solution. The cells were passed through a 70‐μm strainer to avoid cell clumps before detection using a flow cytometer (FACSCanto, BD, USA). Each sample collected contained 2 × 10^4^–20 × 10^4^ cells. Data were analyzed using FlowJo 7.6.1.

### 2.12 LDH Release Test

CD8^+^ T cells and microglia were co‐cultured for 24 h, and the supernatants were collected. Cell damage was detected using an LDH cytotoxicity assay kit (C0016, Beyotime, China). Absorbance was measured at 490 nm with a microplate reader (Model 680, Bio‐Rad, USA) and was positively correlated with LDH activity.

### 2.13 Single‐Cell RNA‐Sequencing (scRNA‐Seq) Analysis

The brainstems of mice were collected and dissociated into single cells after heart perfusion with normal saline. Single‐cell suspensions were loaded onto a 10X Genomics Chromium instrument following the manufacturer's instructions for the 10X Genomics Chromium Single‐Cell 3′ kit (V3). The following cDNA amplification and library construction steps were performed according to the standard protocol. Libraries were sequenced on an Illumina NovaSeq 6000 sequencing system by LC‐Bio Technology Co. Ltd. (China) at a minimum depth of 20,000 reads per cell. A total of 18,647 transcriptomes from single cells in the brainstem of three ECM mice and two healthy mice (minimum number of mice sufficient to provide accurate data analysis) passed the quality control threshold. Cell types were inferred using marker genes identified from the literature and the web‐based tool (http://xteam.xbio.top/CellMarker/). Cell‐to‐cell communication analysis was performed using CellPhoneDB (v3.1.0) based on scRNA‐seq data. Data analysis and graphics generation were performed using the OmicStudio tools at https://www.omicstudio.cn/cell.

### 2.14 Statistical Analysis

Data were processed and analyzed with GraphPad Prism software version 9.5.1. Quantitative data were expressed as mean ± standard deviation. Data normality was assessed using the Shapiro–Wilk test. For two‐group comparisons, normally distributed data were analyzed using Student's t‐test, while non‐normally distributed data were analyzed using the Mann–Whitney U test. Statistical significance was defined as *p* < 0.05.

## 3 Results

### 3.1 Microglia Exhibit a Distinctive Spatial Distribution and Morphological Heterogeneity Within the Nervous Tissue Microenvironment of ECM Brain

**FIGURE 1:**
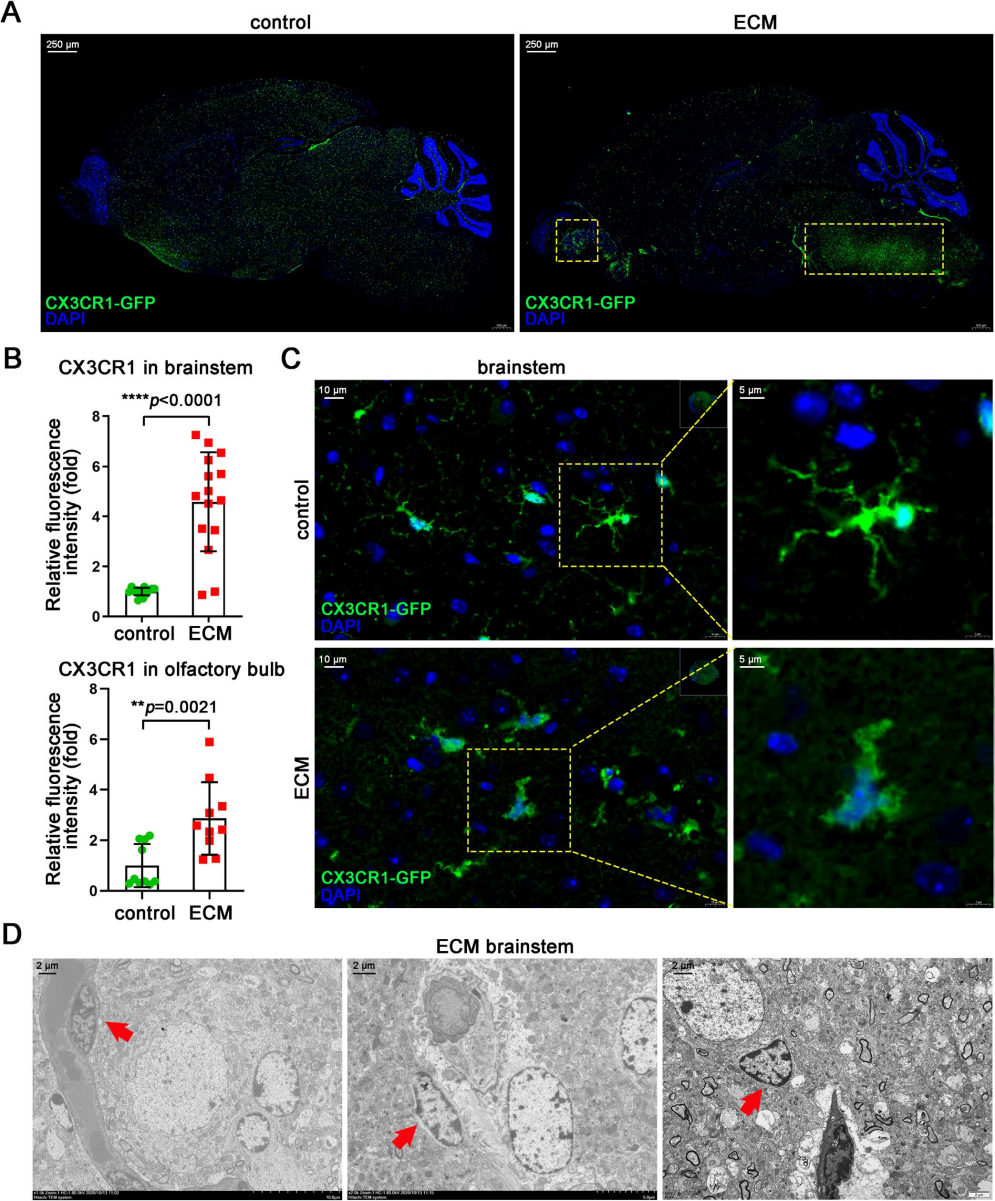
Spatial distribution and morphological heterogeneity of microglia in ECM mice. (A) IF staining of microglia (GFP^+^) in the brains of ECM CX3CR1‐GFP transgenic mice, with uninfected mice as control. (B) Statistical graph of the relative fluorescence intensity of CX3CR1‐GFP in brainstem and olfactory bulb, respectively. Data are expressed as mean ± SD; Mann–Whitney U test; *n* = 10–15 fields per group. (C) IF staining of microglia (GFP^+^) in the brainstems of ECM CX3CR1‐GFP transgenic mice, with uninfected mice as control. (D) Electron microscope observation of microglia (red arrow) in the different parts of ECM brainstem.

In ECM mouse models, neuropathological alterations are characterized by microvascular obstruction, hemorrhages, and increased expression of inflammatory factors, further marked by the presence of activated microglia and infiltrated leukocytes, particularly CD8^+^ T cells [4, 42]. The ECM model, utilizing the CX3CR1‐GFP mice in which microglia were labeled with EGFP in the brain, consistently exhibited neurological symptoms commencing on day 6 post‐infection and died on day 7. H&E staining revealed prominent hemorrhagic spots within the brainstem and olfactory bulb of ECM mice, with the brainstem exhibiting more severe hemorrhaging and a heightened degree of BBB damage, while these phenomena were nearly absent in the brains of control mice (Figure S1A). Nissl staining revealed neuronal damage across all brain regions in ECM mice, particularly in the brainstem (Figure S1B). Correspondingly, the density of microglia in the brainstem and olfactory bulb was significantly elevated in ECM mice compared to that observed in control mice (Figures 1A,B and S1C). These microglia exhibit morphological heterogeneity, featuring enlarged cell bodies and retracted branches compared to the resting microglia in the control group, indicating activation and heightened reactivity (Figure 1C). To detect the spatial localization of microglia in brain lesions of ECM mice, transmission electron microscopy scanning of the brainstem was performed, revealing the presence of microglia situated in proximity to cerebral microvessels, nerve cells, and edematous tissues (Figure 1D).

### 3.2 scRNA‐Seq Analysis Reveals Transcriptional Heterogeneity of ECM Microglia, Distinguishing From Physiological Counterparts

To delve deeper into the state disparities and molecular characteristics of microglia in ECM mice, we conducted single‐cell RNA‐sequencing (scRNA‐seq) on the brainstem of ECM mice with the most severe damage, using the brainstem of uninfected mice as a control group. A total of 11 distinct cell types were identified using cell marker molecules, with astrocytes being the most prevalent, followed closely by microglia, endothelial cells, and oligodendrocytes in descending order of abundance (Figure S2A). The proportion of cells in ECM mice and control mice was different. The proportion of astrocytes and vascular endothelial cells in ECM mice was significantly reduced, indicating nerve cell death and BBB injury, while a significant increase in the proportion of T cells and monocytes indicated immune cell infiltration (Figure S2B).

**FIGURE 2:**
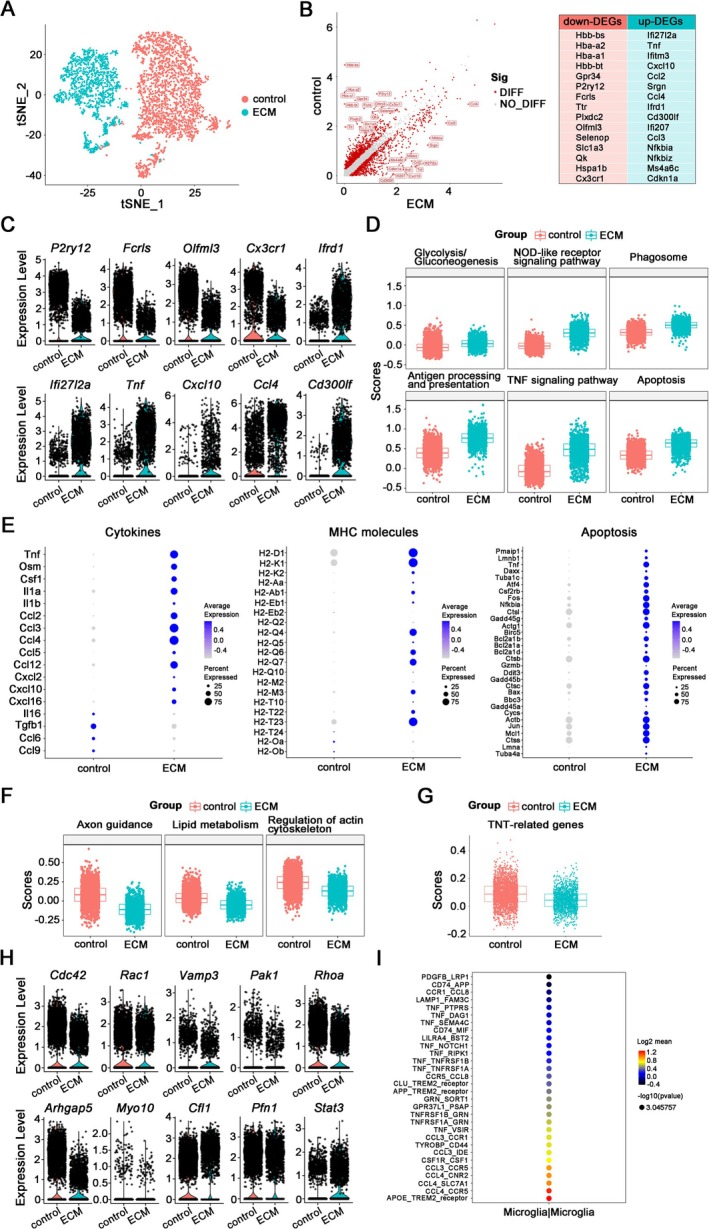
scRNA‐seq analysis of transcriptional profiles of microglia in the brainstem of ECM and control mice. (A) tSNE plots of microglia from ECM (*n* = 3, the same below) and control (*n* = 2, the same below) mice. (B) Volcano plot of top 30 differentially expressed genes (DEGs) in microglia between ECM and control mice. (C) Violin plots of partial DEGs in microglia between ECM and control mice. (D) Gene set scores of activation and apoptosis related genes in microglia. (E) Dotplots of cytokines, MHC molecules, and apoptosis‐related genes in microglia. (F) Gene set scores of axonal guidance, lipid metabolism, and regulation of actin cytoskeleton‐related genes in microglia. (G) Gene set scores of tunneling nanotube (TNT)‐related genes in microglia. (H) Violin plots of TNT‐related genes in microglia. (I) Dotplot of molecular pairs of interaction between microglia of the ECM brainstems.

Through dimensionality reduction clustering, ECM microglia displayed distinct transcriptional signatures that markedly differentiated them from their counterparts in control mice (Figure 2A). There were 2,411 differentially expressed genes in ECM and control microglia, among which 987 genes were up‐regulated and 1,424 genes down‐regulated in ECM microglia (Figure 2B). Among the Top 30 differentially expressed genes, homeostasis‐related genes (*P2ry12*, *Fcrls*, *Olfml3*, *Cx3cr1*) were significantly down‐regulated in ECM microglia compared to control microglia. Conversely, there was a significant upregulation of interferon‐related genes (*Ifrd1*, *Ifi27l2a*), pro‐inflammatory cytokine (*Tnf*) and chemokines (*Cxcl10*, *Ccl4*), as well as phagocytosis‐related genes (*Cd300lf*) (Figure 2C). The gene set scores and Kyoto Encyclopedia of Genes and Genomes (KEGG) pathway analysis of ECM up‐regulated genes in microglia revealed a remarkable upregulation involved in multiple pathways, including glycolysis/gluconeogenesis, NOD‐like receptor signaling pathway, phagosomes, antigen processing and presentation, TNF signaling pathway, and apoptosis in ECM microglia (Figure 2D, Figure S2C). In addition, *Csf1*, pro‐inflammatory cytokines *Il1a* and *Il1b*, as well as MHC‐I and MHC‐II molecules, which play an important regulatory role in the differentiation, polarization, and recruitment of macrophages, were all up‐regulated (Figure 2E). These results underscore the activation of diverse cellular processes in ECM microglia, ranging from metabolic adaptations to enhanced immune responses and programmed cell death, all of which contribute to the intricate pathogenesis of ECM.

However, KEGG pathway analysis for ECM down‐regulated genes combined with gene set scores suggested down‐regulated signaling pathways in ECM microglia, including axonal guidance, endocytosis, lysosome, regulation of actin cytoskeleton, endoplasmic reticulum protein processing, and lipid metabolism (glycerophospholipid, ether lipids, glycerol lipid, etc.) (Figures 2F and S2E). Interestingly, the score of the gene set related to regulation of actin cytoskeleton and tunneling nanotubes (TNTs) production in ECM microglia decreased (Figure 2F,G), and *Cdc42*, *Rac1*, *Vamp3*, *Pak1*, *Rhoa*, *Arhgap5*, *Myo10*, *Stat3*, and other genes associated with TNT production were down‐regulated (Figure 2H). At the same time, *Cfl1*, *Pfn1*, and other molecules that promote F‐actin cleavage and depolymerization were up‐regulated (Figure 2H). In addition, analysis of cell‐to‐cell communication revealed close communication and interaction between microglia (Figures 2I and S2G).

### 3.3 Infiltrating CD8 ^+^ T Cells in the ECM Brainstem Exhibit Sustained Activation Characteristics Associated With Interactions With Microglia

**FIGURE 3:**
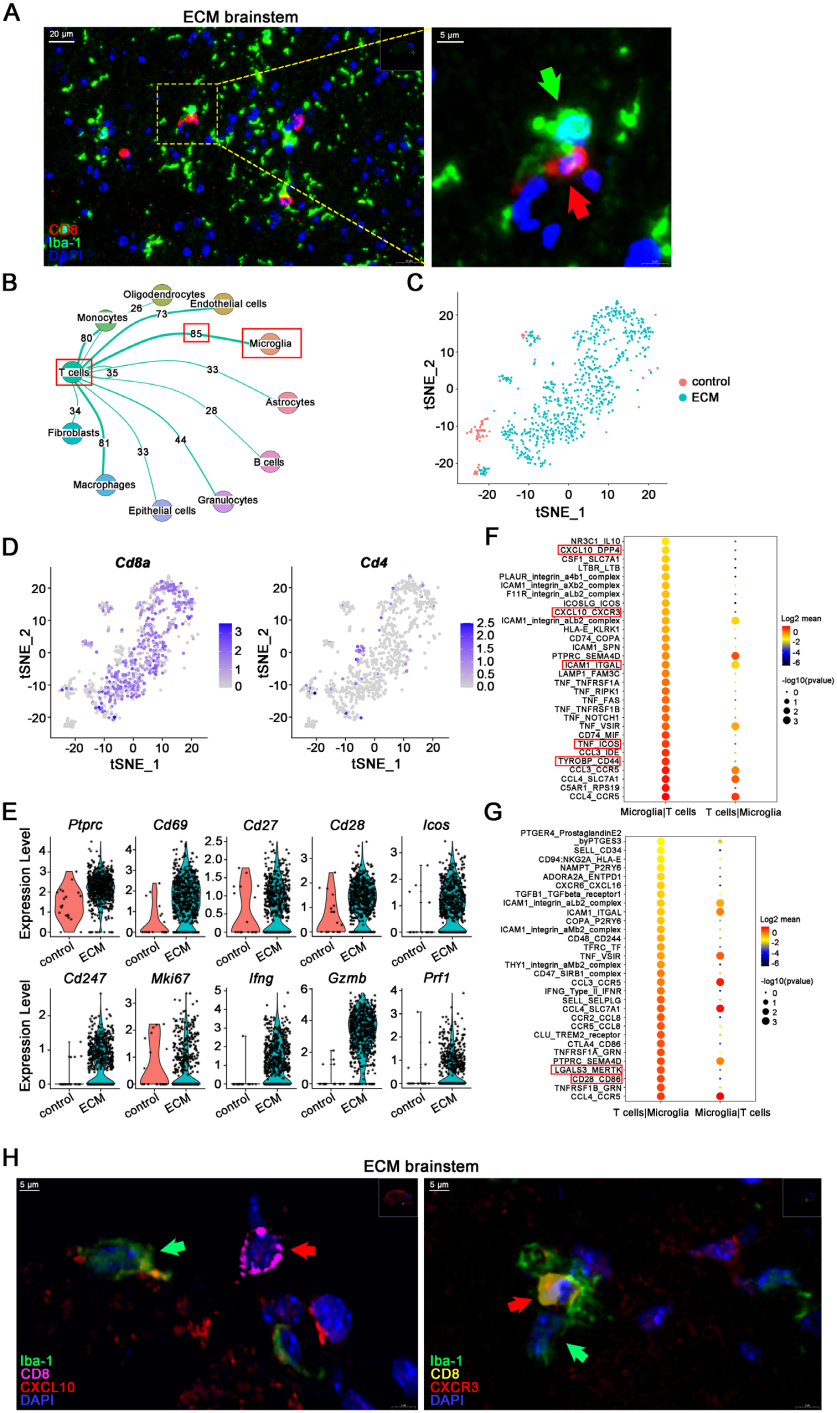
Activated effector phenotype of infiltrating CD8^+^ T cells and close interaction with microglia in the ECM brainstems. (A) IF staining of CD8^+^ T cells (red arrow) and microglia (Iba‐1^+^, green arrow) in the ECM brainstems. (B) Network plot of cell‐to‐cell communication among T cells and other cell types of the ECM brainstems. (C) tSNE plot of T cells in the brainstems of ECM and control mice. (D) tSNE plots of *Cd8a* and *Cd4* expression in T cells of the ECM and control brainstems. (E) Violin plots of activated effector‐related genes in CD8^+^ T cells. (F) Dotplot of molecular pairs of interaction between microglia and T cells of the ECM brainstems. (G) Dotplot of molecular pairs of interaction between T cells and microglia of the ECM brainstems. (H) IF staining of CXCL10 (left) and CXCR3 (right) expression in microglia (green arrow) and CD8^+^ T cell (red arrow) in the ECM brainstems.

A significant number of infiltrating CD8^+^ T cells were found in the brainstem of ECM mice through IF staining, which were spatially close to or even in direct contact with microglia (Figure 3A). Notably, our analysis of single‐cell sequencing data unveiled that the T cells infiltrating the brain exhibited an exceptionally high degree of cell‐to‐cell communication and interaction with microglia (Figure 3B). Consistent with our previous findings [11], the number and proportion of T cells in the brainstem of ECM mice were much higher than those of control mice, with unique transcriptional characteristics (Figures 3C and S2B). Through further analysis of marker molecules, the overwhelming majority of T cells residing within the brainstem of ECM mice were indeed CD8^+^ T cells (Figure 3D). These infiltrative ECM CD8^+^ T cells have upregulated activation markers such as *Ptprc*, *Cd69*, *Cd27*, *Cd28*, *Icos*, *Ifng*, CD3/TCR complex component *Cd247*, cell proliferation‐related genes *Mki67*, and cytotoxic effector molecules *Gzmb*, *Prf1*, among others (Figure 3E), indicating CD8^+^ T cells in the ECM brain displayed an activated effector cell phenotype. Furthermore, chemotactic pairs between CD8^+^ T cells and microglia exhibited a strong pattern of interaction. Molecular pairs of interaction between microglia and infiltrating T cells were listed based on cell‐to‐cell communication analysis. Microglia recruited (*Cxcl10*‐*Cxcr3*, *Cxcl10*‐*Dpp4*), adhered to (*Icam1*‐*Itgal*), and activated (*Tyrobp*‐*Cd44*, *Tnf*‐*Icos*, *Cd28*‐*Cd86*) CD8^+^ T cells (Figure 3F,G); CD8^+^ T cells stimulated microglial phagocytic pathways (*Lgals3*‐*Mertk*) (Figure 3G). IF staining verified the spatial proximity between CXCL10^+^ microglia and CXCR3^+^CD8^+^ T cells in ECM brainstems (Figure 3H).

### 3.4 Subcluster Analysis Reveals a Subtype of ECM‐Associated Microglia Associated With T Cell Recruitment and Sustained Activation

**FIGURE 4:**
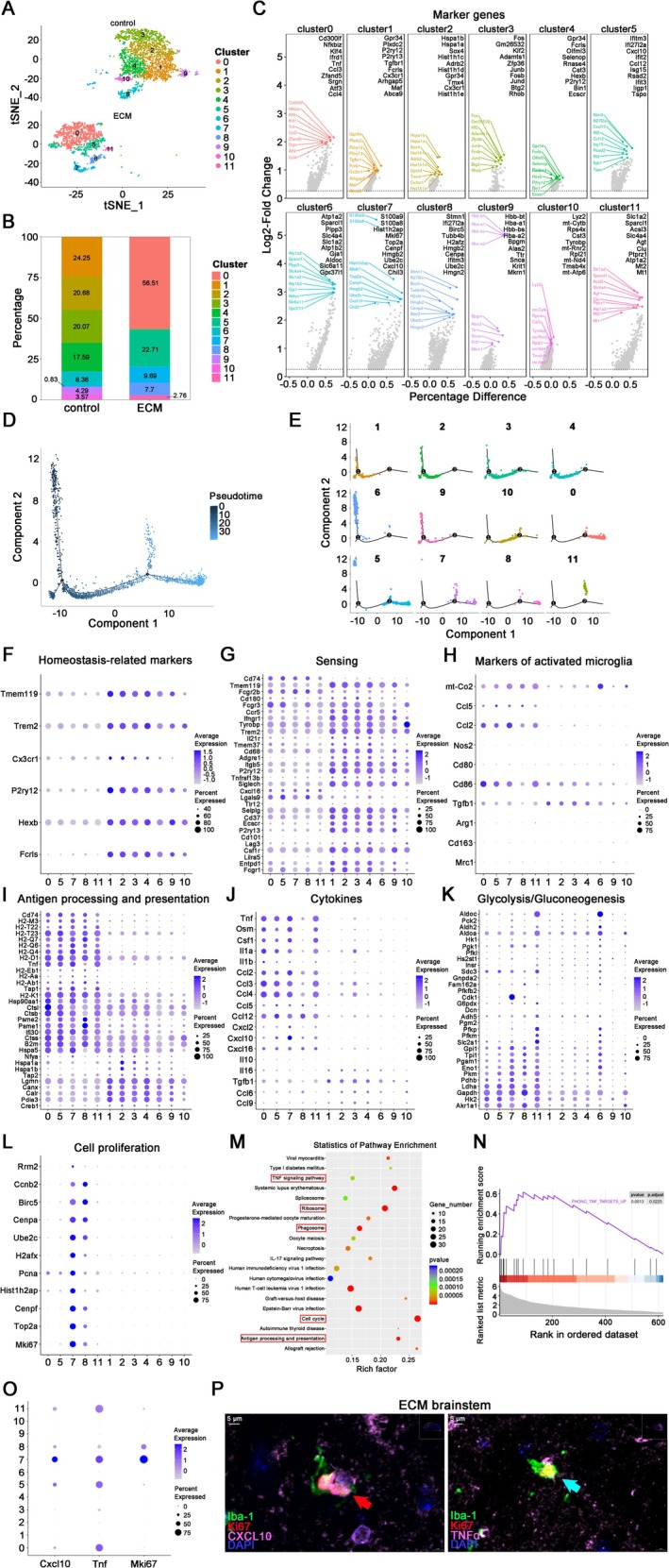
Subcluster analysis of microglia in the brainstem of ECM and control mice. (A) UMAP plot of clusters of microglia in ECM and control mice. (B) Proportion of different clusters of microglia. (C) Top 10 marker genes of different clusters of microglia. (D) Pseudotime ordering of all microglia in ECM and control mice. (E) Pseudotime ordering presented by microglia clusters. (F–L) Dotplots of genes related to homeostasis markers (F), sensing (G), activated microglia markers (H), antigen processing and presentation (I), cytokines (J), glycolysis/gluconeogenesis (K), and cell proliferation (L) in different clusters of microglia. (M) KEGG pathway enrichment analysis of cluster 7 microglia. (N) GSEA analysis of TNF‐related genes in cluster 7 microglia. (O) Dotplot of *Mki67*, *Cxcl10*, and *Tnf* expression in different clusters of microglia. (P) IF staining of CXCL10^+^Ki67^+^ microglia (red arrow) and TNFα^+^Ki67^+^ microglia (blue arrow) in the ECM brainstems.

To further reveal the molecular characteristics of different clusters, we conducted subcluster analysis on data of 4,334 microglia. Microglia were composed of 12 transcriptional clusters with unique marker genes in control and ECM mice (Figure 4A,C). Cluster 0/5/7/8/11 mainly existed in ECM mice, and the remaining clusters mainly existed in control mice (Figure 4A,B). The size of different clusters varied greatly. Cluster 0 accounted for 56.51% of all microglia, while cluster 11 accounted for only 2.76% (Figure 4B).

To elucidate the switching between transcriptional states, we analyzed scRNA‐seq data using Monocle 2 [43]. By implementing pseudotime ordering analysis of the entire microglial population, a comprehensive trajectory consisting of a total of five distinct states categorized into two major branches (Figure 4D). Combined with other molecular characteristics, clusters 1/2/3/4/9/10 could be preliminarily identified as mature resting microglia, and their homeostasis‐related markers and sensing genes responsible for continuously sensing environmental changes were expressed higher than those in other clusters (Figure 4E–G). Cluster 6 represented immature microglia in early development, with lower expression of homeostasis‐related markers and sensing genes than in other resting clusters, while the glycolysis/gluconeogenesis‐related genes were higher (Figure 4E–G,K). Clusters 0/5/7/8/11 were activated microglia, with clusters 5/7/8/11 in the early activation stage, while cluster 0 was in the late activation stage (Figure 4D–F,H). The antigen processing and presentation and pro‐inflammatory cytokine related genes of cluster 0 were higher than those in other activated microglia (Figure 4I,J). It is worth noting that cluster 0 had the largest number of cells among all clusters (Figure 4C). These findings indicate that most microglia in the brainstem were in a state of inflammatory activation, characterized by enhanced phagocytosis and increased secretion of cytokines, following BBB injury during the late stages of PbA infection.

Among the microglia clusters activated during ECM pathogenesis, cluster 7 was a noteworthy ECM‐associated subtype, and its unique activation characteristics were obviously related to the recruitment and activation of CD8^+^ T cells, suggesting a pivotal role in modulating the adaptive immune response within the ECM microenvironment. In addition to the general characteristics of activated microglia, the expression of the CD8^+^ T cell chemokine CXCL10 and genes related to cell proliferation were significantly higher in this cluster compared to other clusters (Figure 4J,L). KEGG pathway enrichment analysis of cluster 7 microglia revealed that up‐regulated pathways were enriched in cell cycle, antigen processing and presentation, ribosome, phagosome, and the TNF signaling pathway (Figure 4M). Gene set enrichment analysis (GSEA) indicated a significant enrichment of TNF‐related genes in cluster 7 microglia (Figure 4N). Based on the characteristics of cluster 7, the ECM‐associated microglia were characterized by CXCL10^high^TNFα^high^Ki67^+^ (Figure 4O). Correspondingly, we found CXCL10^+^Ki67^+^ microglia and TNFα^+^Ki67^+^ microglia in the brainstem of ECM mice, respectively (Figure 4P). The transcriptional characteristics of ECM‐associated microglia display a remarkable degree of heterogeneity. Notably, the CXCL10^high^TNFα^high^Ki67^+^ microglia exhibit a highly activated and proliferative phenotype, intimately linked to the recruitment and activation of CD8^+^ T cells within the brain.

### 3.5 Co‐Stimulation of TNFα, IFNγ, and pRBC Triggers CXCL10^high^TNFα^high^Ki67 ^+^ Phenotype Expression in Primary Cultured Microglia

**FIGURE 5:**
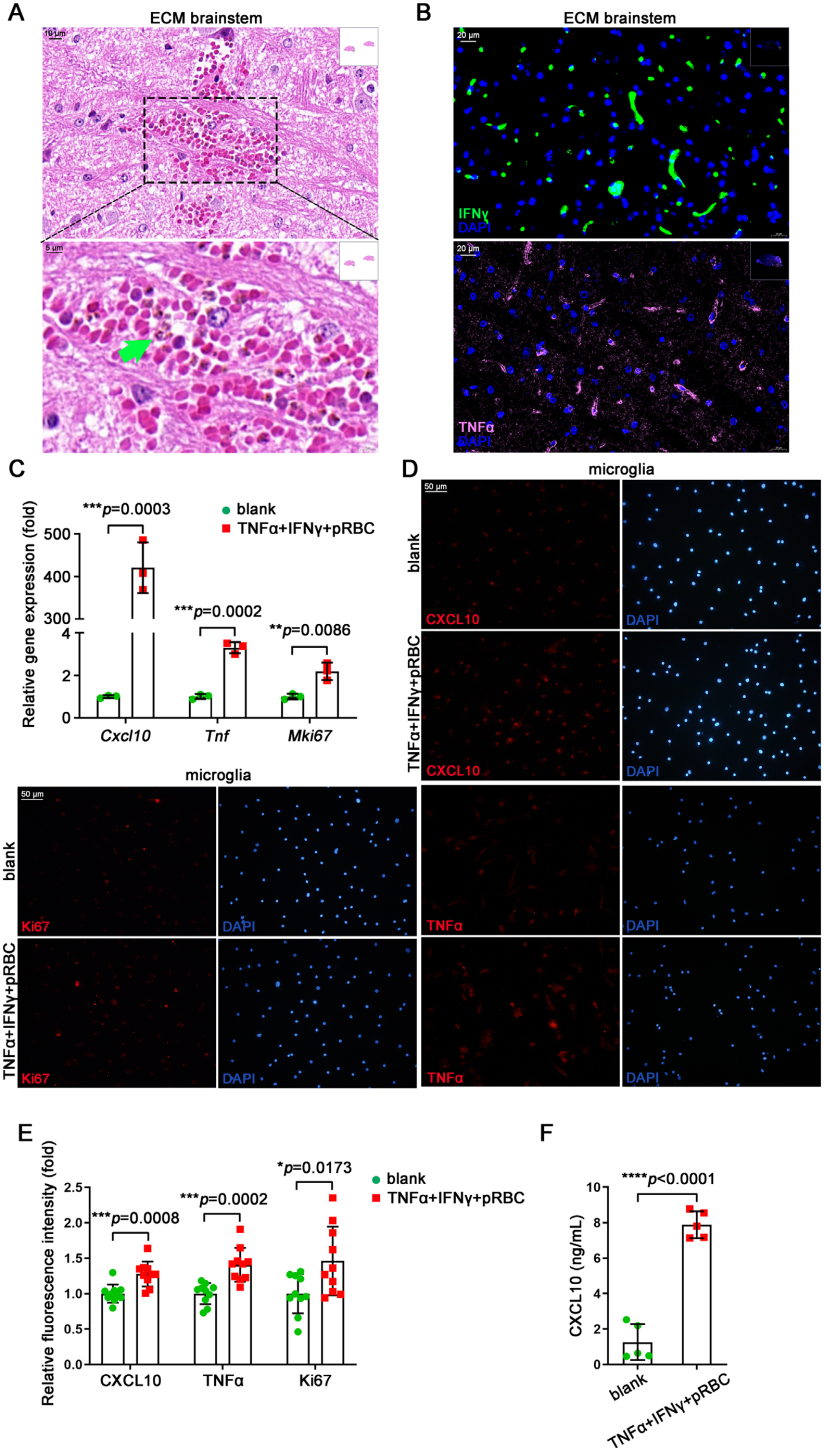
Inducing CXCL10^high^TNFα^high^Ki67^+^ microglia in vitro. (A) H&E staining of hemorrhagic spot in the brainstem of ECM mice (green arrow: pRBC). (B) IF staining of IFNγ and TNFα in the brainstem of ECM mice. (C) q‐PCR detection of *Cxcl10*, *Tnf*, and *Mki67* expression in primary microglia with co‐stimulation of TNFα (10 ng/mL, the same below), IFNγ (10 ng/mL, the same below), and pRBC (pRBC: Microglia = 20: 1, the same below) for 12 h. Data are expressed as mean ± SD; unpaired t‐test; *n* = 3. (D) IF staining of CXCL10, TNFα, and Ki67 in microglia with co‐stimulation for 24 h. (E) Statistical graph of the relative fluorescence intensity in Figure 5D. Data are expressed as mean ± SD; unpaired t‐test; *n* = 10 fields per group. (F) ELISA detection of CXCL10 in culture supernatants of microglia with co‐stimulation for 24 h. Data are expressed as mean ± SD; unpaired t‐test; *n* = 5.

The pRBCs have been observed to enter the brainstem of ECM mice after the BBB damage and may be recognized and engulfed by microglia according to H&E staining (Figure 5A). Many studies have confirmed the crucial role of inflammatory cytokines, such as IFNγ and TNFα, in the development of ECM. The widespread expression of IFNγ and TNFα was also revealed by IF staining in the brainstem of ECM mice (Figure 5B). In order to observe whether the CXCL10^high^TNFα^high^Ki67^+^ microglia exist under simulated brain immune microenvironment of ECM mice in vitro, we stimulated primary microglia with the combination of TNFα, IFNγ, and pRBC. Utilizing q‐PCR detection, ECM‐associated microglia marker genes *Cxcl10*, *Tnf*, and *Mki67* were significantly up‐regulated after co‐stimulation for 12 h (Figure 5C), and the same results were obtained in the microglia cell line BV2 (Figure S3A). IF staining also showed elevated levels of CXCL10 and TNFα and increased Ki67^+^ microglia after co‐stimulation for 24 h (Figure 5D,E). Meanwhile, the level of CXCL10 secreted in the cell culture supernatant was also significantly up‐regulated after co‐stimulation for 24 h by ELISA detection (Figure 5F). These results indicated that under simulated conditions of the brain immune microenvironment in ECM mice, primary microglia showed the same immune signatures as those identified by scRNA‐seq, further confirming the existence of CXCL10^high^TNFα^high^Ki67^+^ microglia closely related to ECM.

### 3.6 CXCL10^high^TNFα^high^Ki67 ^+^ Microglia Recruit ECM‐Activated CD8 ^+^ T Cells

**FIGURE 6:**
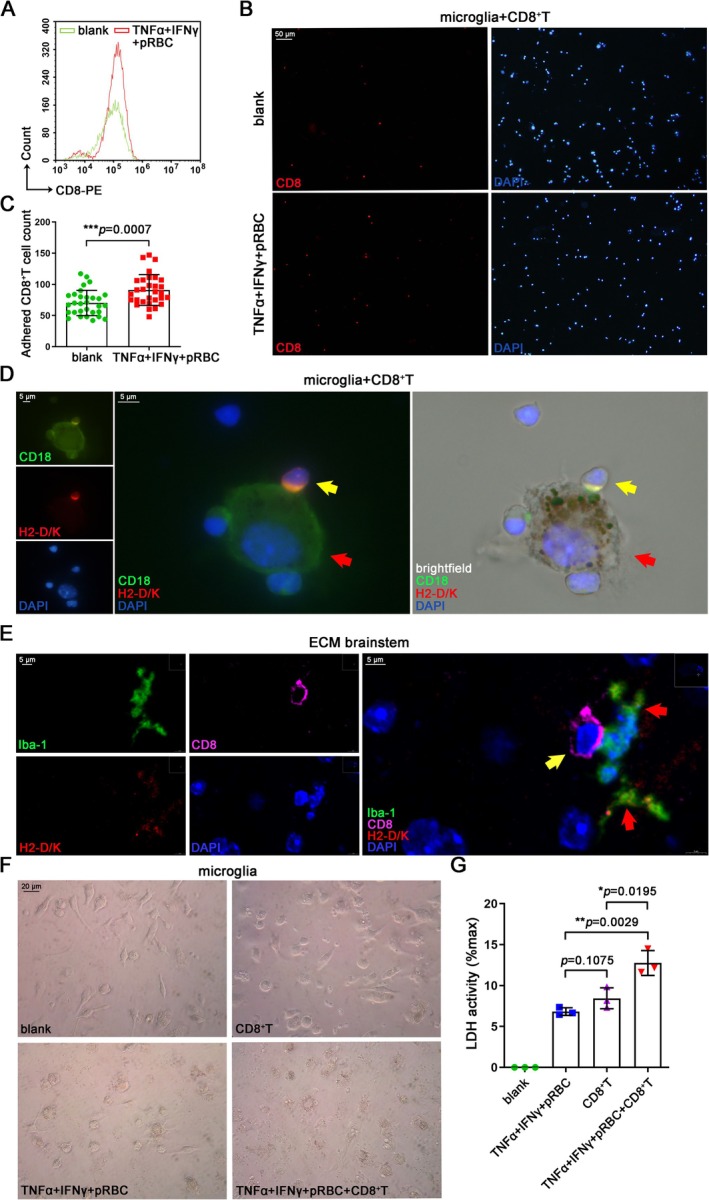
Interaction between CXCL10^high^TNFα^high^Ki67^+^microglia and ECM‐activated CD8^+^ T cells in vitro. (A) FC detection of CD8^+^ T cells recruited in the lower chamber of Transwell system, with CXCL10^high^TNFα^high^Ki67^+^ microglia cultured in the lower chamber. (B) IF staining of adhered CD8^+^ T cells after co‐culture with CXCL10^high^TNFα^high^Ki67^+^ microglia for 24 h. (C) Statistical graph of the adhered CD8^+^ T cell count in Figure 6B. Data are expressed as mean ± SD; unpaired t‐test; *n* = 30 fields per group. (D) IF staining of CD18 and H2‐D/K after co‐culture of CD8^+^ T cells (yellow arrow) and CXCL10^high^TNFα^high^Ki67^+^ microglia (red arrow) for 4 h. (E) IF staining of H2‐D/K in microglia (red arrow) and CD8^+^ T cell (yellow arrow) in the ECM brainstems. (F) Optical microscope observation of the CXCL10^high^TNFα^high^Ki67^+^ microglia co‐cultured with CD8^+^ T cells for 24 h. (G) LDH relative activity in supernatants of CXCL10^high^TNFα^high^Ki67^+^ microglia co‐cultured with CD8^+^ T cells for 24 h. Data are expressed as mean ± SD; unpaired t‐test; *n* = 3.

To investigate the interaction between CXCL10^high^TNFα^high^Ki67^+^ microglia and CD8^+^ T cells, microglia were inoculated and cultured in the lower chamber of the Transwell system, while CD8^+^ T cells isolated from the spleen of ECM mice were added to the upper chamber. After 4 h of culture, cells from the lower chamber were collected and subjected to FC analysis. The results showed that CXCL10^high^TNFα^high^Ki67^+^ microglia were able to recruit more CD8^+^ T cells (Figure 6A). Similarly, IF staining showed more CD8^+^ T cells recruited in the lower chamber of the Transwell system, with CXCL10^high^ TNFα^high^ Ki67^+^ BV2 cultured in the lower chamber (Figure S4A,B). In addition, microglia were co‐cultured with CD8^+^ T cells for 24 h, and it was shown that CXCL10^high^TNFα^high^Ki67^+^ microglia could adhere to more CD8^+^ T cells based on IF staining (Figure 6B,C). IF staining also showed co‐localization of the co‐stimulatory cell‐surface molecule CD18 (LFA‐1) and the MHC‐I molecule H2‐D/K [44], suggesting that activated microglia and CD8^+^ T cells may form an “immune synapse‐like” structure (Figure 6D), which was also observed between activated BV2 and CD8^+^ T cells (Figure S4C). Similarly, in ECM brainstems, we observed close contacts between H2‐D/K^+^ microglia and CD8^+^ T cells (Figure 6E). Observation under an optical microscope revealed that CXCL10^high^TNFα^high^Ki67^+^ microglia devoured a large amount of malarial pigment and the cell morphology changed, with larger cell bodies and fewer branches (Figure 6D,F). After the addition of CD8^+^ T cells, microglia adhered to and were damaged by CD8^+^ T cells (Figure 6F). Furthermore, the LDH assay revealed a pronounced exacerbation of cell damage in CXCL10^high^TNFα^high^Ki67^+^ microglia upon co‐cultivation with CD8^+^ T cells (Figure 6G), while the same results were obtained in BV2 (Figure S4D,E). Notably, even in the absence of direct co‐stimulation or when CD8^+^ T cells were present alone, a measurable level of cellular injury was observed, albeit to a lesser extent. These results indicated that CXCL10^high^TNFα^high^Ki67^+^ microglia could recruit CD8^+^ T cells in vitro to form “immune synapse‐like” structures.

### 3.7 CXCL10^high^TNFα^high^Ki67 ^+^ Microglia Maintain Sustained Activation of CD8 ^+^ T Cells Through Persistent Interactions

**FIGURE 7:**
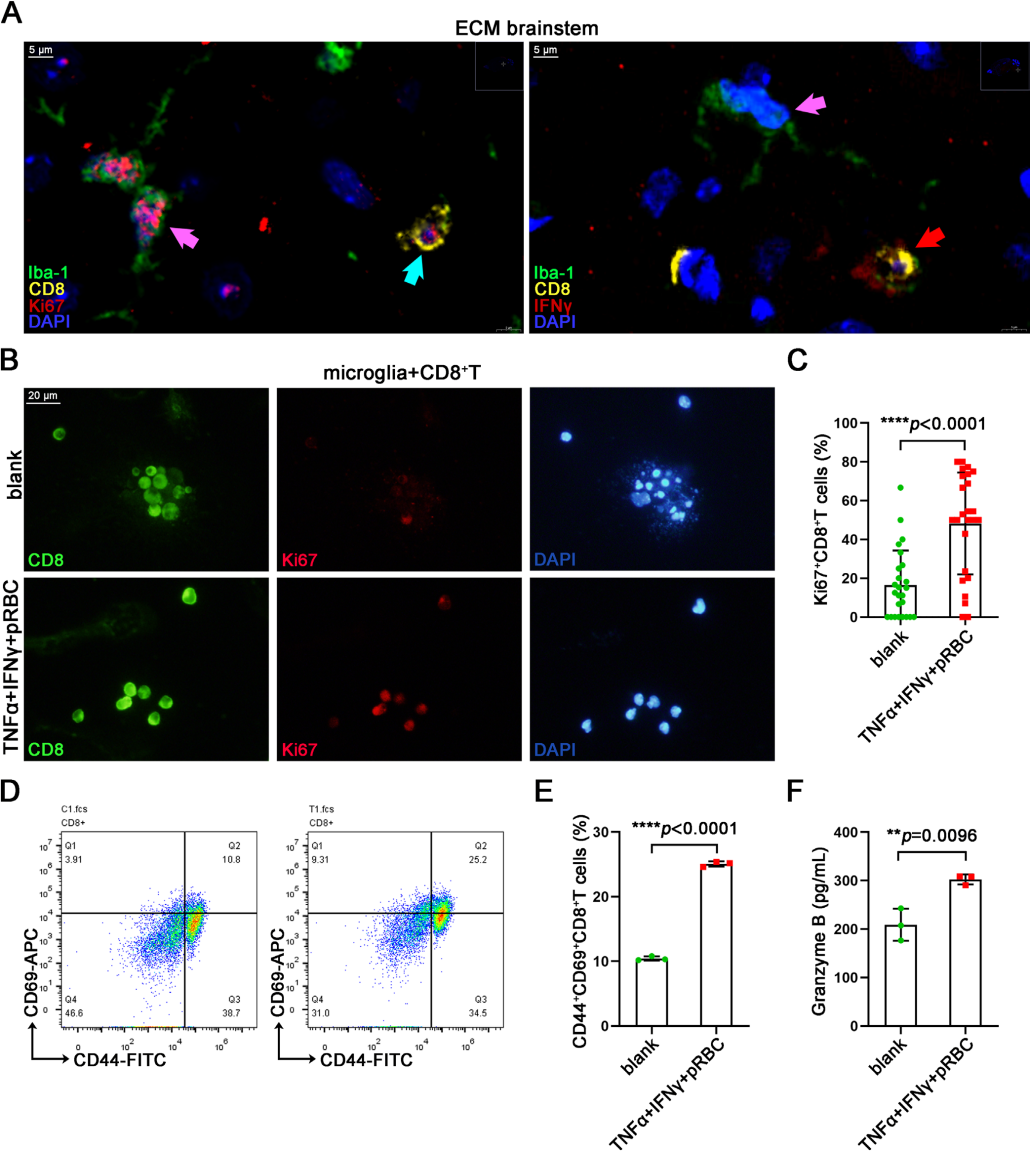
CXCL10^high^TNFα^high^Ki67^+^ microglia continuously activate CD8^+^ T cells in vitro. (A) IF staining of Ki67^+^CD8^+^ T cell (blue arrow) and IFNγ^+^CD8^+^ T cell (red arrow) adjacent to microglia (pink arrow) in the ECM brainstems. (B) IF staining of ki67^+^CD8^+^ T cells after co‐culture with CXCL10^high^TNFα^high^Ki67^+^ microglia for 24 h. (C) Statistical graph of the proportion of ki67^+^CD8^+^ T cells in CD8^+^ T cells in Figure 7B. Data are expressed as mean ± SD; Mann–Whitney U test; *n* = 25 fields per group. (D) FC detection of CD44^+^CD69^+^CD8^+^ T cells after co‐culture with CXCL10^high^TNFα^high^Ki67^+^ microglia for 24 h. (E) Statistical graph of the proportion of CD44^+^CD69^+^CD8^+^ T cells in CD8^+^ T cells in Figure 7D. Data are expressed as mean ± SD; unpaired t‐test; *n* = 3. (F) ELISA detection of granzyme B in co‐culture supernatants of CXCL10^high^TNFα^high^Ki67^+^ microglia and CD8^+^ T cells for 24 h. Data are expressed as mean ± SD; unpaired t‐test; *n* = 3.

Sustained activation of CD8^+^ T cells in the brain parenchyma is essential for the development of CM neuroinflammation and brain injury [33]. In the above experiments, we showed that microglia could recruit CD8^+^ T cells and form “immune synapse‐like” structures with them. However, the question remains unanswered whether this intricate interaction directly fosters the sustained activation of CD8^+^ T cells. IF staining of the brainstem in ECM mice revealed that CD8^+^ T cells adjacent to microglia exhibit proliferative activity (Ki67^+^) and secrete the effector cytokine IFNγ, indicating a highly activated state (Figure 7A). Similarly, the CD8^+^ T cells showed a higher proportion of Ki67^+^ cells after co‐culture with CXCL10^high^TNFα^high^Ki67^+^ microglia compared to those co‐cultured with unstimulated microglia through IF staining (Figure 7B,C). The FC analysis also indicated an increase in the proportion of CD44^+^CD69^+^CD8^+^ T cells after co‐culture with CXCL10^high^TNFα^high^Ki67^+^ microglia (Figures 7D,E and S5A–C). The detection of culture supernatant showed that CD8^+^ T cells co‐cultured with CXCL10^high^TNFα^high^Ki67^+^ microglia secreted more cytotoxic effector granzyme B (Figure 7F). These results indicated that CXCL10^high^TNFα^high^Ki67^+^ microglia could continuously activate CD8^+^ T cells in vitro to maintain their proliferative activity and cytotoxic function.

## 4 Discussion

As resident immune cells of the CNS, microglia swiftly respond to pathological alterations and fluctuations in the immune microenvironment [45]. This response encompasses rapid proliferation, migration towards the site of injury or lesion, phagocytosis of pathogens and cellular debris, as well as the production and secretion of cytokines and chemokines [18]. Studies have definitively indicated that in the brains of ECM mice, microglial activation precedes the onset of neurological symptoms by a significant window of 2–3 days [38, 39]. Understanding the role of microglia and their interaction with other immune cells in the pathogenesis of CM could lead to the development of new therapeutic strategies. In this study, using scRNA‐seq analysis, we compared the transcriptional characteristics and functional differences between ECM and resting microglia. Based on subcluster analysis, we obtained a total of 12 distinct transcriptional clusters and further identified a subtype of ECM‐associated microglia characterized by CXCL10^high^TNFα^high^Ki67^+^, with proliferative activity, pro‐inflammatory effect, as well as the ability of recruiting and continuously activating CD8^+^ T cells to aggravate neuropathology in ECM, which were confirmed by in vitro experiments.

The specific activation phenotype of microglial cells, situated within the unique CM immune microenvironment, intricately dictates their functional roles. We found increased density and morphological changes of microglia in the brainstem of ECM mice. At the transcriptome level, ECM microglia exhibited unique transcriptional characteristics, with down‐regulation of microglia homeostatic genes and upregulation of activated genes related to glycolysis, pro‐inflammatory cytokines and chemokines, phagosomes, antigen processing and presentation. However, except for cluster 7 and a small number of cluster 8 microglia, the rest of microglia did not show proliferation characteristics. These two clusters account for only a small proportion (17.39%) of ECM microglia and may be at the early stages of activation. The samples for our scRNA‐seq were from mice infected with PbA for 7 days, at which point the mice were already in the late stage of ECM with severe BBB disruption. This may be the reason for the low number of cells in proliferative clusters 7 and 8, and the highest number of cells in cluster 0 with the final activation phase.

In our investigation, we uncovered an ECM‐associated microglia type distinguished by its profile of CXCL10^high^TNFα^high^Ki67^+^. Specifically, Ki67^+^ signifies heightened proliferative activity, a hallmark of activated microglia, which underscores their involvement in the neuroinflammatory response. TNFα, predominantly secreted by macrophages, plays an important role in orchestrating the inflammatory cascade within the brain. Its presence in the CNS could elicit detrimental effects such as vasoconstriction, disruption of tissue homeostasis, and BBB impairment [46]. Notably, elevated TNFα levels have been implicated in the severity of CM, with survivors exhibiting twofold higher plasma TNFα levels compared to uncomplicated malaria patients, and deceased cases showing 10‐fold increases [47]. Furthermore, our focus on CXCL10 reveals its pivotal contribution to CM pathogenesis. CXCL10, emanating from activated endothelial cells, acts as a potent chemokine that attracts CXCR3‐expressing CD8^+^ T cells [48, 49, 50]. This recruitment process fosters the accumulation of cytotoxic CD8^+^ T cells within the brains of CM patients, contributing to immunopathology [6, 51]. In humans, elevated serum CXCL10 levels correlate strongly with CM‐related mortality [52, 53], whereas CXCL10 neutralization in ECM mouse models has been shown to confer protection [54]. Our current study adds a novel dimension to the CXCL10 narrative by revealing that microglia are among the sources of CXCL10 within the brain parenchyma. This finding suggests a mechanism whereby CXCL10, under the guidance of microglial secretion, actively recruits CD8^+^ T cells into the brain parenchyma, thereby exacerbating ECM‐associated neuropathology.

Distinct from other neuroinflammation and neurodegeneration diseases, which primarily rely on the activation of CD4^+^ T cells [8, 10], CM uniquely orchestrates a dominant role for CD8^+^ T cells in its immune response. Nevertheless, the intricate mechanisms underpinning this CD8^+^ T cell dominance in CM remain largely uncharted territories of scientific inquiry. In such a context, this study delves deeper into the intricate interplay between CD8^+^ T cells and the neuroimmune environment in ECM, particularly focusing on the activation and effector functions of these cells within the brain parenchyma. CD8^+^ T cells need to re‐recognize the antigen within the brain to elicit cytotoxic activity, a process facilitated by cerebral vascular endothelial cells that cross‐present *Plasmodium* antigens to CD8^+^ T cells, initiating the so‐called intravascular CD8^+^ T cell activation [29]. Our investigation further elucidates the sustained activation of CD8^+^ T cells post‐BBB disruption and their subsequent infiltration into the brain parenchyma. Notably, CXCL10^high^TNFα^high^Ki67^+^ microglia characterized by heightened expression of MHC‐I molecules engage in immune synapse‐like interactions with CD8^+^ T cells. These interactions facilitate the continuous activation of CD8^+^ T cells, prompting them to release cytotoxic effector molecules that target and eliminate microglia. While direct evidence of microglial cross‐presentation of *Plasmodium* antigens to CD8^+^ T cells remains elusive in our current study, other existing research, both in vitro and in vivo, underscores the capacity of microglia to cross‐present antigens [37, 55, 56]. This implies that microglia, under certain conditions, could potentially present *Plasmodium* antigens to CD8^+^ T cells, thereby contributing to the perpetuation of the inflammatory cascade within the brain.

In this study, we successfully induced CXCL10^high^TNFα^high^Ki67^+^ microglia in vitro through a triple stimulation protocol that enabled us to study their behavior under conditions mimicking the pathological milieu of ECM. However, more research on microglia in different pathological and physiological conditions has demonstrated that microglial heterogeneity extends beyond the temporal dimension, exhibiting regional variability as well [57, 58]. Distinct brain regions contain microglia that exhibit significantly divergent gene expression profiles, morphologies, and functional capabilities [57, 58]. These differences are likely due to the unique neural architectures, connectivity patterns, and localized microenvironments present in each region. Crucially, in the context of neurological disorders, microglia undergo specific pathological transformations, encompassing alterations in cell morphology, the secretion of inflammatory mediators, and intricate interactions with other immune components [59, 60]. Therefore, in follow‐up experiments, we can perform spatial transcriptomics analysis [61] of samples at different disease stages of ECM mice that allow visualization and quantitative analysis of the transcriptome with spatial resolution, and enrich the library of microglia subtypes and their corresponding molecular characteristics, while focusing on the characteristics of ECM‐associated microglia.

## 5 Conclusions

Overall, our study delved into the intricate heterogeneity and distinct features of microglia in ECM mice through scRNA‐seq analysis and experiments. Notably, we uncovered a unique subtype of ECM‐associated microglia characterized by a robust expression profile of CXCL10^high^TNFα^high^Ki67^+^. This particular subtype of microglia plays a pivotal role in recruiting CD8^+^ T cells into the brain parenchyma and maintaining their sustained activation. The infiltrating CD8^+^ T cells, in turn, contribute to the exacerbation of ECM neuropathology by mediating nerve cell killing. This finding offers new insights into the potential of developing novel immunotherapy strategies that target this critical interaction between microglia and T cells.

## Author Contributions

Conceptualization and methodology: Y.W., J.L., Y.S., J.W., Q.Z., G.T., G.L., and Y.Z.; Investigation: Y.W., J.L., C.Y., G.T., and T.L.; Formal analysis: Y.W., C.Y., Q.Z., T.L., and Y.Y.; Resources: Y.Z., J.R., J.L., Y.S., J.W., and Y.L.; Writing – Original draft preparation: Y.W., Y.S., J.L., and C.Y.; Visualization: Y.W., C.Y., G.T., and G.L.; Supervision: Y.Z., J.L., J.R., Y.S., J.W., Y.H., and Y.L.; Writing – Reviewing and Editing: Y.Z. and Y.S.; All authors read and approved the final manuscript.

## Ethics Statement

The Institutional Review Board of the Air Force Medical University (No. IACUC‐20200407) approved animal experiments. All procedures were performed to minimize injury to the experimental mice.

## Conflicts of Interest

The authors declare no conflicts of interest.

## Supporting information


**Figure S1.** BBB injury in ECM mice.
**Figure S2.** scRNA‐seq analysis of microglia in the brainstem of ECM and control mice.
**Figure S3.** Inducing CXCL10^high^TNFα^high^Ki67^+^ BV2 in vitro.
**Figure S4.** Interaction between CXCL10^high^TNFα^high^Ki67^+^ BV2 and CD8^+^ T cells in vitro.
**Figure S5.** Sustained activation of CD8^+^ T cells in vitro detected by flow cytometry.
**Table S1.** Primers used for q‐PCR in this study.

## Data Availability

Data sets generated and analyzed during the current study are available from the corresponding author upon reasonable request. Our scRNA‐seq data under the BioProject ID: PRJNA735877 have been submitted to the database of the NCBI Sequence Read Archive (http://www.ncbi.nlm.nih.gov/bioproject/).

## References

[1] P. Venkatesan , “The 2023 WHO World Malaria Report,” Lancet Microbe 5, no. 3 (2024): e214, 10.1016/S2666-5247(24)00016-8. 38309283

[2] A. M. Dondorp , S. J. Lee , M. A. Faiz , et al., “The Relationship Between Age and the Manifestations of and Mortality Associated With Severe Malaria,” Clinical Infectious Diseases 47, no. 2 (2008): 151–157, 10.1086/589287. 18533842

[3] A. L. Conroy , R. O. Opoka , P. Bangirana , et al., “Parenteral Artemisinins Are Associated With Reduced Mortality and Neurologic Deficits and Improved Long‐Term Behavioral Outcomes in Children With Severe Malaria,” BMC Medicine 19, no. 1 (2021): 168, 10.1186/s12916-021-02033-1. 34315456 PMC8317420

[4] A. Hadjilaou , J. Brandi , M. Riehn , M. A. Friese , and T. Jacobs , “Pathogenetic Mechanisms and Treatment Targets in Cerebral Malaria,” Nature Reviews. Neurology 19, no. 11 (2023): 688–709, 10.1038/s41582-023-00881-4. 37857843

[5] J. Nitcheu , O. Bonduelle , C. Combadiere , et al., “Perforin‐Dependent Brain‐Infiltrating Cytotoxic CD8+ T Lymphocytes Mediate Experimental Cerebral Malaria Pathogenesis,” Journal of Immunology 170, no. 4 (2003): 2221–2228, 10.4049/jimmunol.170.4.2221. 12574396

[6] B. A. Riggle , M. Manglani , D. Maric , et al., “CD8+ T Cells Target Cerebrovasculature in Children With Cerebral Malaria,” Journal of Clinical Investigation 130, no. 3 (2020): 1128–1138, 10.1172/JCI133474. 31821175 PMC7269583

[7] S. P. Kurup , N. S. Butler , and J. T. Harty , “T Cell‐Mediated Immunity to Malaria,” Nature Reviews. Immunology 19, no. 7 (2019): 457–471, 10.1038/s41577-019-0158-z. PMC6599480 30940932

[8] D. Gate , E. Tapp , O. Leventhal , et al., “CD4+ T Cells Contribute to Neurodegeneration in Lewy Body Dementia,” Science 374, no. 6569 (2021): 868–874, 10.1126/science.abf7266. 34648304 PMC9122025

[9] M. Charabati , S. Zandee , A. P. Fournier , et al., “MCAM+ Brain Endothelial Cells Contribute to Neuroinflammation by Recruiting Pathogenic CD4+ T Lymphocytes,” Brain 146, no. 4 (2023): 1483–1495, 10.1093/brain/awac389. 36319587 PMC10115172

[10] S. X. Shi , Y. Xiu , Y. Li , et al., “CD4+ T Cells Aggravate Hemorrhagic Brain Injury,” Science Advances 9, no. 23 (2023): eabq0712, 10.1126/sciadv.abq0712. 37285421 PMC10246900

[11] J. Wang , Q. Zhu , Y. Shen , et al., “CD8+ T Cell Infiltration and Proliferation in the Brainstem During Experimental Cerebral Malaria,” CNS Neuroscience & Therapeutics 30, no. 3 (2024): e14431, 10.1111/cns.14431. 37697956 PMC10916431

[12] M. Prinz and J. Priller , “Microglia and Brain Macrophages in the Molecular Age: From Origin to Neuropsychiatric Disease,” Nature Reviews. Neuroscience 15, no. 5 (2014): 300–312, 10.1038/nrn3722. 24713688

[13] L. Fourgeaud , P. G. Través , Y. Tufail , et al., “TAM Receptors Regulate Multiple Features of Microglial Physiology,” Nature 532, no. 7598 (2016): 240–244, 10.1038/nature17630. 27049947 PMC5358512

[14] C. Sousa , K. Biber , and A. Michelucci , “Cellular and Molecular Characterization of Microglia: A Unique Immune Cell Population,” Frontiers in Immunology 8 (2017): 198, 10.3389/fimmu.2017.00198. 28303137 PMC5332364

[15] Q. Li , Z. Cheng , L. Zhou , et al., “Developmental Heterogeneity of Microglia and Brain Myeloid Cells Revealed by Deep Single‐Cell RNA Sequencing,” Neuron 101, no. 2 (2019): 207–223, 10.1016/j.neuron.2018.12.006. 30606613 PMC6336504

[16] R. Masgrau , C. Guaza , R. M. Ransohoff , and E. Galea , “Should we Stop Saying “Glia” and “Neuroinflammation”?,” Trends in Molecular Medicine 23, no. 6 (2017): 486–500, 10.1016/j.molmed.2017.04.005. 28499701

[17] M. Colonna and O. Butovsky , “Microglia Function in the Central Nervous System During Health and Neurodegeneration,” Annual Review of Immunology 35 (2017): 441–468, 10.1146/annurev-immunol-051116-052358. PMC8167938 28226226

[18] T. R. Hammond , C. Dufort , L. Dissing‐Olesen , et al., “Single‐Cell RNA Sequencing of Microglia Throughout the Mouse Lifespan and in the Injured Brain Reveals Complex Cell‐State Changes,” Immunity 50, no. 1 (2019): 253–271, 10.1016/j.immuni.2018.11.004. 30471926 PMC6655561

[19] G. J. Harry , “Microglia During Development and Aging,” Pharmacology & Therapeutics 139, no. 3 (2013): 313–326, 10.1016/j.pharmthera.2013.04.013. 23644076 PMC3737416

[20] A. Silvin , S. Uderhardt , C. Piot , et al., “Dual Ontogeny of Disease‐Associated Microglia and Disease Inflammatory Macrophages in Aging and Neurodegeneration,” Immunity 55, no. 8 (2022): 1448–1465.e6, 10.1016/j.immuni.2022.07.004. 35931085

[21] C. Madore , Z. Yin , J. Leibowitz , and O. Butovsky , “Microglia, Lifestyle Stress, and Neurodegeneration,” Immunity 52, no. 2 (2020): 222–240, 10.1016/j.immuni.2019.12.003. 31924476 PMC7234821

[22] A. Mary , R. Mancuso , and M. T. Heneka , “Immune Activation in Alzheimer Disease,” Annual Review of Immunology 42, no. 1 (2024): 585–613, 10.1146/annurev-immunol-101921-035222. 38424470

[23] A. Mantovani , A. Sica , and M. Locati , “Macrophage Polarization Comes of Age,” Immunity 23, no. 4 (2005): 344–346, 10.1016/j.immuni.2005.10.001. 16226499

[24] E. Z. Macosko , A. Basu , R. Satija , et al., “Highly Parallel Genome‐Wide Expression Profiling of Individual Cells Using Nanoliter Droplets,” Cell 161, no. 5 (2015): 1202–1214, 10.1016/j.cell.2015.05.002. 26000488 PMC4481139

[25] H. Mathys , C. Adaikkan , F. Gao , et al., “Temporal Tracking of Microglia Activation in Neurodegeneration at Single‐Cell Resolution,” Cell Reports 21, no. 2 (2017): 366–380, 10.1016/j.celrep.2017.09.039. 29020624 PMC5642107

[26] H. Keren‐Shaul , A. Spinrad , A. Weiner , et al., “A Unique Microglia Type Associated With Restricting Development of Alzheimer's Disease,” Cell 169, no. 7 (2017): 1276–1290, 10.1016/j.cell.2017.05.018. 28602351

[27] T. Masuda , R. Sankowski , O. Staszewski , et al., “Spatial and Temporal Heterogeneity of Mouse and Human Microglia at Single‐Cell Resolution,” Nature 566, no. 7744 (2019): 388–392, 10.1038/s41586-019-0924-x. 30760929

[28] J. Dunst , F. Kamena , and K. Matuschewski , “Cytokines and Chemokines in Cerebral Malaria Pathogenesis,” Frontiers in Cellular and Infection Microbiology 7 (2017): 324, 10.3389/fcimb.2017.00324. 28775960 PMC5517394

[29] S. W. Howland , C. M. Poh , S. Y. Gun , et al., “Brain Microvessel Cross‐Presentation Is a Hallmark of Experimental Cerebral Malaria,” EMBO Molecular Medicine 5, no. 7 (2013): 984–999, 10.1002/emmm.201202273. 23681698 PMC3721469

[30] S. W. Howland , C. Claser , C. M. Poh , S. Y. Gun , and L. Rénia , “Pathogenic CD8+ T Cells in Experimental Cerebral Malaria,” Seminars in Immunopathology 37, no. 3 (2015): 221–231, 10.1007/s00281-015-0476-6. 25772948

[31] V. Barrera , M. J. Haley , P. Strangward , et al., “Comparison of CD8+ T Cell Accumulation in the Brain During Human and Murine Cerebral Malaria,” Frontiers in Immunology 10 (2019): e1747, 10.3389/fimmu.2019.01747. PMC6668485 31396236

[32] T. N. Shaw , P. J. Stewart‐Hutchinson , P. Strangward , et al., “Perivascular Arrest of CD8+ T Cells Is a Signature of Experimental Cerebral Malaria,” PLoS Pathogens 11, no. 11 (2015): e1005210, 10.1371/journal.ppat.1005210. 26562533 PMC4643016

[33] Y. Wang , Y. Shen , J. Liang , et al., “Neurons Upregulate PD‐L1 via IFN/STAT1/IRF1 to Alleviate Damage by CD8+ T Cells in Cerebral Malaria,” Journal of Neuroinflammation 21, no. 1 (2024): 119, 10.1186/s12974-024-03114-7. 38715061 PMC11077882

[34] J. Liang , Y. Shen , Y. Wang , et al., “Ferroptosis Participates in Neuron Damage in Experimental Cerebral Malaria and Is Partially Induced by Activated CD8+ T Cells,” Molecular Brain 15, no. 1 (2022): 57, 10.1186/s13041-022-00942-7. 35725567 PMC9208218

[35] P. A. Sutter and S. J. Crocker , “Glia as Antigen‐Presenting Cells in the Central Nervous System,” Current Opinion in Neurobiology 77 (2022): 102646, 10.1016/j.conb.2022.102646. 36371828 PMC10183975

[36] X. Chen , M. Firulyova , M. Manis , et al., “Microglia‐Mediated T Cell Infiltration Drives Neurodegeneration in Tauopathy,” Nature 615, no. 7953 (2023): 668–677, 10.1038/s41586-023-05788-0. 36890231 PMC10258627

[37] E. A. Moseman , A. C. Blanchard , D. Nayak , and D. B. McGavern , “T Cell Engagement of Cross‐Presenting Microglia Protects the Brain From a Nasal Virus Infection,” Science Immunology 5, no. 48 (2020): eabb1817, 10.1126/sciimmunol.abb1817. 32503876 PMC7416530

[38] B. Capuccini , J. Lin , C. Talavera‐López , et al., “Transcriptomic Profiling of Microglia Reveals Signatures of Cell Activation and Immune Response, During Experimental Cerebral Malaria,” Scientific Reports 6 (2016): 39258, 10.1038/srep39258. 27991544 PMC5171943

[39] I. M. Medana , N. H. Hunt , and T. Chan‐Ling , “Early Activation of Microglia in the Pathogenesis of Fatal Murine Cerebral Malaria,” Glia 19, no. 2 (1997): 91–103, 10.1002/(sici)1098-1136(199702)19:2<91::aid-glia1>3.0.co;2-c. 9034826

[40] I. M. Medana , T. Chan‐Ling , and N. H. Hunt , “Reactive Changes of Retinal Microglia During Fatal Murine Cerebral Malaria: Effects of Dexamethasone and Experimental Permeabilization of the Blood‐Brain Barrier,” American Journal of Pathology 156, no. 3 (2000): 1055–1065, 10.1016/S0002-9440(10)64973-5. 10702421 PMC1876828

[41] Y. Shen , Y. Li , Q. Zhu , et al., “The Immunomodulatory Effect of Microglia on ECM Neuroinflammation via the PD‐1/PD‐L1 Pathway,” CNS Neuroscience & Therapeutics 28, no. 1 (2022): 46–63, 10.1111/cns.13760. 34766463 PMC8673706

[42] C. Coban , M. S. J. Lee , and K. J. Ishii , “Tissue‐Specific Immunopathology During Malaria Infection,” Nature Reviews. Immunology 18, no. 4 (2018): 266–278, 10.1038/nri.2017.138. PMC7097228 29332936

[43] X. Qiu , Q. Mao , Y. Tang , et al., “Reversed Graph Embedding Resolves Complex Single‐Cell Trajectories,” Nature Methods 14, no. 10 (2017): 979–982, 10.1038/nmeth.4402. 28825705 PMC5764547

[44] J. Lötscher , I. Martí , A. A. Líndez , et al., “Magnesium Sensing via LFA‐1 Regulates CD8+ T Cell Effector Function,” Cell 185, no. 4 (2022): 585–602, 10.1016/j.cell.2021.12.039. 35051368

[45] M. Prinz , S. Jung , and J. Priller , “Microglia Biology: One Century of Evolving Concepts,” Cell 179, no. 2 (2019): 292–311, 10.1016/j.cell.2019.08.053. 31585077

[46] N. R. Sibson , A. M. Blamire , V. H. Perry , J. Gauldie , P. Styles , and D. C. Anthony , “TNF‐Alpha Reduces Cerebral Blood Volume and Disrupts Tissue Homeostasis via an Endothelin‐ and TNFR2‐Dependent Pathway,” Brain 125, no. Pt 11 (2002): 2446–2459, 10.1093/brain/awf256. 12390971

[47] D. Kwiatkowski , A. V. Hill , I. Sambou , et al., “TNF Concentration in Fatal Cerebral, Non‐Fatal Cerebral, and Uncomplicated Plasmodium Falciparum Malaria,” Lancet 336, no. 8725 (1990): 1201–1204, 10.1016/0140-6736(90)92827-5. 1978068

[48] G. Nishanth and D. Schlüter , “Blood‐Brain Barrier in Cerebral Malaria: Pathogenesis and Therapeutic Intervention,” Trends in Parasitology 35, no. 7 (2019): 516–528, 10.1016/j.pt.2019.04.010. 31147271

[49] G. S. V. Campanella , A. M. Tager , J. K. El Khoury , et al., “Chemokine Receptor CXCR3 and Its Ligands CXCL9 and CXCL10 Are Required for the Development of Murine Cerebral Malaria,” Proceedings of the National Academy of Sciences of the United States of America 105, no. 12 (2008): 4814–4819, 10.1073/pnas.0801544105. 18347328 PMC2290783

[50] J. Miu , A. J. Mitchell , M. Müller , et al., “Chemokine Gene Expression During Fatal Murine Cerebral Malaria and Protection due to CXCR3 Deficiency,” Journal of Immunology 180, no. 2 (2008): 1217–1230, 10.4049/jimmunol.180.2.1217. 18178862

[51] E. W. Sorensen , J. Lian , A. J. Ozga , et al., “CXCL10 Stabilizes T Cell‐Brain Endothelial Cell Adhesion Leading to the Induction of Cerebral Malaria,” JCI Insight 3 (2018): e98911. 29669942 10.1172/jci.insight.98911 PMC5931132

[52] N. O. Wilson , V. Jain , C. E. Roberts , et al., “CXCL4 and CXCL10 Predict Risk of Fatal Cerebral Malaria,” Disease Markers 30, no. 1 (2011): 39–49, 10.3233/DMA-2011-0763. 21508508 PMC3260027

[53] H. B. Armah , N. O. Wilson , B. Y. Sarfo , et al., “Cerebrospinal Fluid and Serum Biomarkers of Cerebral Malaria Mortality in Ghanaian Children,” Malaria Journal 6 (2007): 147, 10.1186/1475-2875-6-147. 17997848 PMC2186349

[54] C. Q. Nie , N. J. Bernard , M. U. Norman , et al., “IP‐10‐Mediated T Cell Homing Promotes Cerebral Inflammation Over Splenic Immunity to Malaria Infection,” PLoS Pathogens 5, no. 4 (2009): e1000369, 10.1371/journal.ppat.1000369. 19343215 PMC2658824

[55] C. Beauvillain , S. Donnou , U. Jarry , et al., “Neonatal and Adult Microglia Cross‐Present Exogenous Antigens,” Glia 56, no. 1 (2008): 69–77, 10.1002/glia.20565. 17932942

[56] U. Jarry , P. Jeannin , L. Pineau , S. Donnou , Y. Delneste , and D. Couez , “Efficiently Stimulated Adult Microglia Cross‐Prime Naive CD8+ T Cells Injected in the Brain,” European Journal of Immunology 43, no. 5 (2013): 1173–1184, 10.1002/eji.201243040. 23529826

[57] R. Sankowski , P. Süß , A. Benkendorff , et al., “Multiomic Spatial Landscape of Innate Immune Cells at Human Central Nervous System Borders,” Nature Medicine 30, no. 1 (2024): 186–198, 10.1038/s41591-023-02673-1. PMC10803260 38123840

[58] W. T. Chen , A. Lu , K. Craessaerts , et al., “Spatial Transcriptomics and in Situ Sequencing to Study Alzheimer's Disease,” Cell 182, no. 4 (2020): 976–991, 10.1016/j.cell.2020.06.038. 32702314

[59] Q. Li and B. A. Barres , “Microglia and Macrophages in Brain Homeostasis and Disease,” Nature Reviews. Immunology 18, no. 4 (2018): 225–242, 10.1038/nri.2017.125. 29151590

[60] M. W. Salter and B. Stevens , “Microglia Emerge as Central Players in Brain Disease,” Nature Medicine 23, no. 9 (2017): 1018–1027, 10.1038/nm.4397. 28886007

[61] P. L. Ståhl , F. Salmén , S. Vickovic , et al., “Visualization and Analysis of Gene Expression in Tissue Sections by Spatial Transcriptomics,” Science 353, no. 6294 (2016): 78–82, 10.1126/science.aaf2403. 27365449

