# Supplementary material for: CXCL10highTNFαhighKi67 + Microglia Recruit and Activate CD8 + T Cells in the Brainstem During Experimental Cerebral Malaria
Source: CNS Neurosci Ther. 2025 Jun 2;31(6):e70425. doi: 10.1111/cns.70425 (PMC12129709; doi:10.1111/cns.70425)
Supplement: Supplementary file 1 — Figure S1. BBB injury in ECM mice. Figure S2. scRNA‐seq analysis of microglia in the brainstem of ECM and control mice. Figure S3. Inducing CXCL10highTNFαhighKi67+ BV2 in vitro. Figure S4. Interaction between CXCL10highTNFαhighKi67+ BV2 and CD8+ T cells in vitro. Figure S5. Sustained activation of CD8+ T cells in vitro detected by flow cytometry. Table S1. Primers used for q‐PCR in this study. [file CNS-31-e70425-s001.pdf]

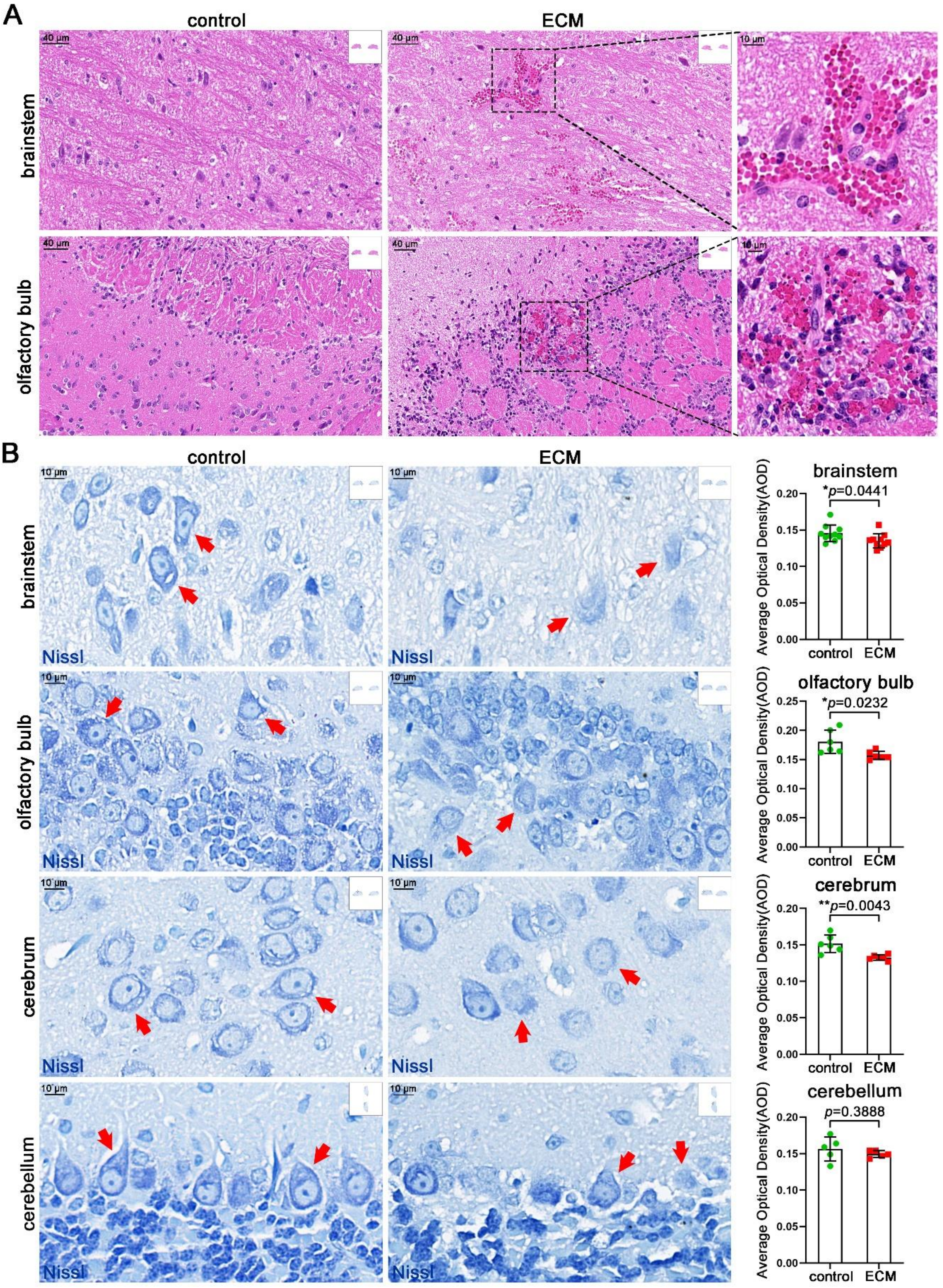

A
control
ECM
brainstem
olfactory bulb
40 μm
10 μm
B
control
ECM
brainstem
olfactory bulb
cerebrum
cerebellum
Nissl
10 μm
brainstem
*p=0.0441
olfactory bulb
*p=0.0232
cerebrum
**p=0.0043
cerebellum
p=0.3888
Average Optical Density(AOD)
control
ECM

C

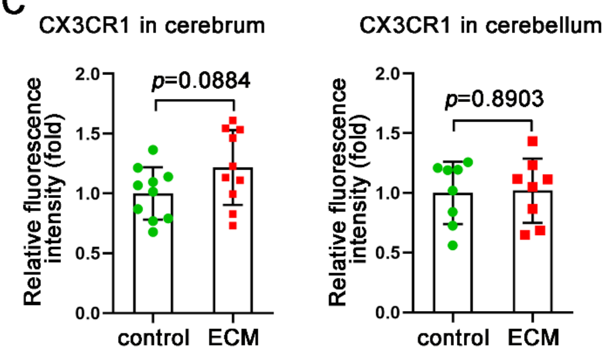

Fig. S1 BBB injury and neuron damage in ECM mice. (**A**) H&E staining of hemorrhagic spot in the brainstem and olfactory bulb of ECM mice. (**B**) Nissl staining of neurons in brainstem, olfactory bulb, cerebrum, and cerebellum of ECM mice. Data are expressed as mean ± SD; unpaired t-test; n = 5-10 fields per group. (**C**) Statistical graph of relative fluorescence intensity of CX3CR1-GFP in cerebrum and cerebellum, respectively. Data are expressed as mean ± SD; unpaired t-test; n = 8-10 fields per group.

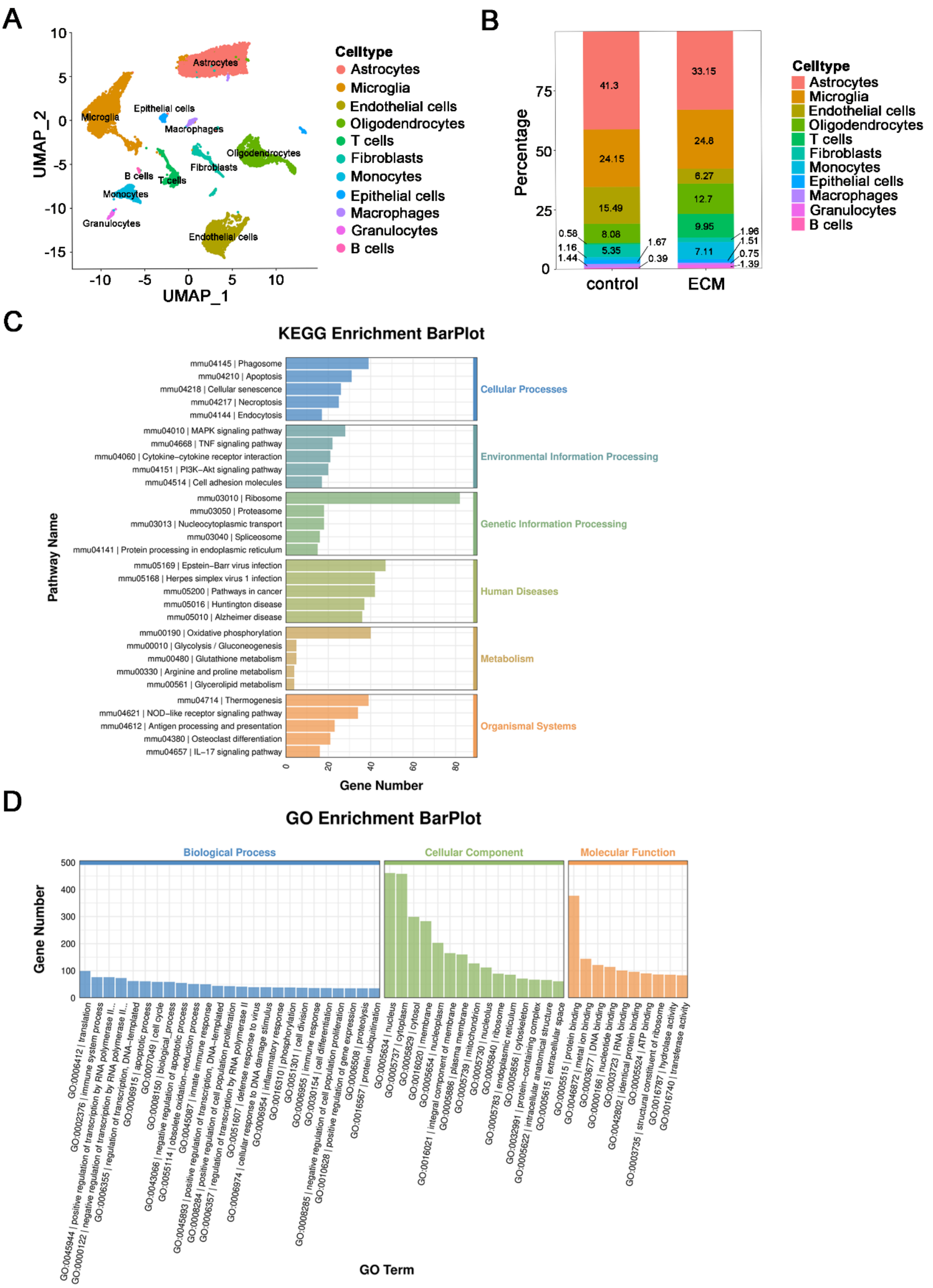

A
UMAP_2
UMAP_1
Astrocytes
Microglia
Epithelial cells
Macrophages
Oligodendrocytes
Fibroblasts
B cells
T cells
Monocytes
Granulocytes
Endothelial cells
Celltype
Astrocytes
Microglia
Endothelial cells
Oligodendrocytes
T cells
Fibroblasts
Monocytes
Epithelial cells
Macrophages
Granulocytes
B cells
B
Percentage
control
ECM
41.3
24.15
15.49
8.08
5.35
0.58
1.16
1.44
1.67
0.39
33.15
24.8
6.27
12.7
9.95
7.11
1.96
1.51
0.75
1.39
Celltype
Astrocytes
Microglia
Endothelial cells
Oligodendrocytes
T cells
Fibroblasts
Monocytes
Epithelial cells
Macrophages
Granulocytes
B cells
C
KEGG Enrichment BarPlot
Pathway Name
mmu04145 | Phagosome
mmu04210 | Apoptosis
mmu04218 | Cellular senescence
mmu04217 | Necroptosis
mmu04144 | Endocytosis
Cellular Processes
mmu04010 | MAPK signaling pathway
mmu04668 | TNF signaling pathway
mmu04060 | Cytokine−cytokine receptor interaction
mmu04151 | PI3K−Akt signaling pathway
mmu04514 | Cell adhesion molecules
Environmental Information Processing
mmu03010 | Ribosome
mmu03050 | Proteasome
mmu03013 | Nucleocytoplasmic transport
mmu03040 | Spliceosome
mmu04141 | Protein processing in endoplasmic reticulum
Genetic Information Processing
mmu05169 | Epstein−Barr virus infection
mmu05168 | Herpes simplex virus 1 infection
mmu05200 | Pathways in cancer
mmu05016 | Huntington disease
mmu05010 | Alzheimer disease
Human Diseases
mmu00190 | Oxidative phosphorylation
mmu00010 | Glycolysis / Gluconeogenesis
mmu00480 | Glutathione metabolism
mmu00330 | Arginine and proline metabolism
mmu00561 | Glycerolipid metabolism
Metabolism
mmu04714 | Thermogenesis
mmu04621 | NOD−like receptor signaling pathway
mmu04612 | Antigen processing and presentation
mmu04380 | Osteoclast differentiation
mmu04657 | IL−17 signaling pathway
Organismal Systems
Gene Number
D
GO Enrichment BarPlot
Gene Number
Biological Process
Cellular Component
Molecular Function
GO:0006412 | translation
GO:0002376 | immune system process
GO:0045944 | positive regulation of transcription by RNA polymerase II...
GO:0000122 | negative regulation of transcription by RNA polymerase II...
GO:0006355 | regulation of transcription, DNA−templated
GO:0006915 | apoptotic process
GO:0007049 | cell cycle
GO:0008150 | biological_process
GO:0043066 | negative regulation of apoptotic process
GO:0055114 | obsolete oxidation−reduction process
GO:0045087 | innate immune response
GO:0045893 | positive regulation of transcription, DNA−templated
GO:0008284 | positive regulation of cell population proliferation
GO:0006357 | regulation of transcription by RNA polymerase II
GO:0051607 | defense response to virus
GO:0006974 | cellular response to DNA damage stimulus
GO:0006954 | inflammatory response
GO:0016310 | phosphorylation
GO:0051301 | cell division
GO:0006955 | immune response
GO:0030154 | cell differentiation
GO:0008285 | negative regulation of cell population proliferation
GO:0010628 | positive regulation of gene expression
GO:0006508 | proteolysis
GO:0016567 | protein ubiquitination
GO:0005634 | nucleus
GO:0005737 | cytoplasm
GO:0005829 | cytosol
GO:0016020 | membrane
GO:0005654 | nucleoplasm
GO:0016021 | integral component of membrane
GO:0005886 | plasma membrane
GO:0005739 | mitochondrion
GO:0005730 | nucleolus
GO:0005840 | ribosome
GO:0005783 | endoplasmic reticulum
GO:0005856 | cytoskeleton
GO:0032991 | protein−containing complex
GO:0005622 | intracellular anatomical structure
GO:0005615 | extracellular space
GO:0005515 | protein binding
GO:0046872 | metal ion binding
GO:0003677 | DNA binding
GO:0000166 | nucleotide binding
GO:0003723 | RNA binding
GO:0042802 | identical protein binding
GO:0005524 | ATP binding
GO:0003735 | structural constituent of ribosome
GO:0016787 | hydrolase activity
GO:0016740 | transferase activity
GO Term

E

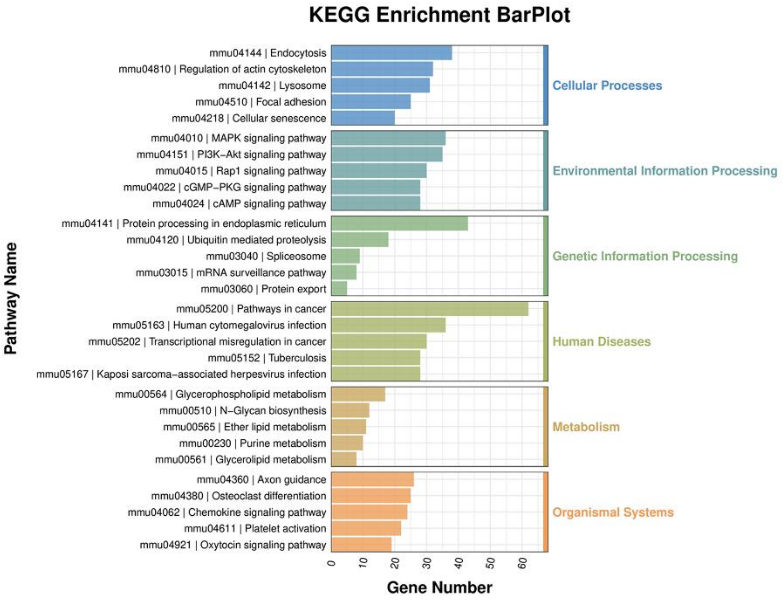

F

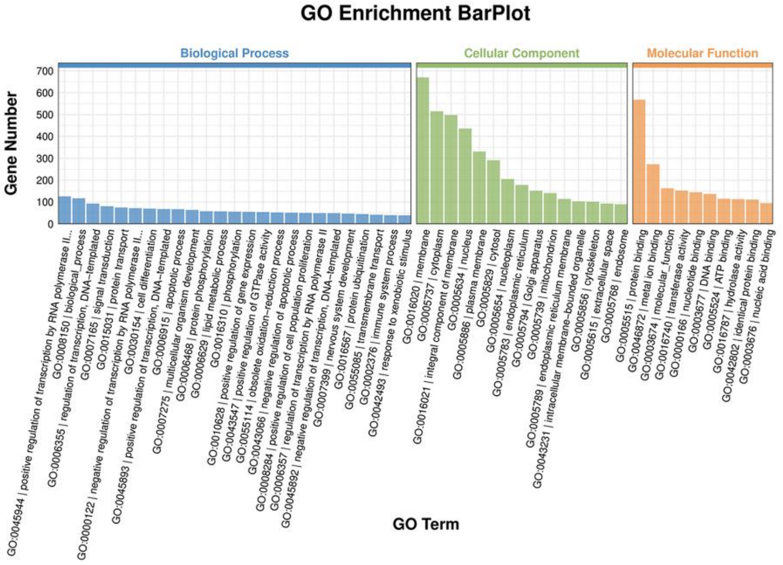

G

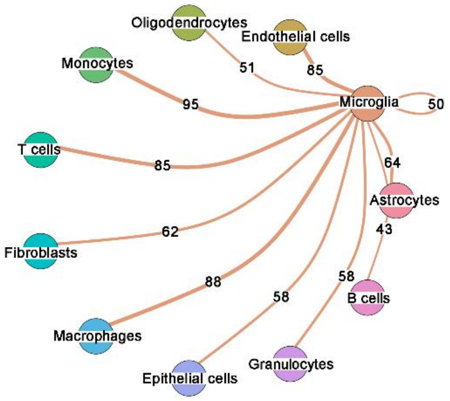

Fig. S2 scRNA-seq analysis of microglia in the brainstem of ECM and control mice. (**A**) UMAP plot of cell types of ECM (n = 3) and control (n = 2) mice. (**B**) Proportion of different cell types of ECM and control mice. (**C**) KEGG enrichment barplot of up-regulated genes in microglia from ECM mice. (**D**) GO enrichment barplot of up-regulated genes in microglia from

ECM mice. (**E**) KEGG enrichment barplot of down-regulated genes in microglia from ECM mice. (**F**) GO enrichment barplot of down-regulated genes in microglia from ECM mice. (**G**) Network plot of cell-to-cell communication among microglia and other cell types of the ECM brainstems.

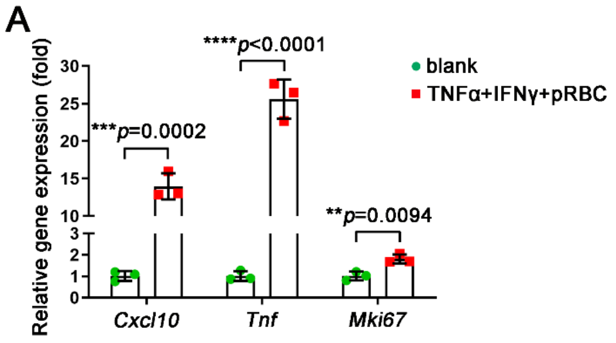

Fig. S3 Inducing CXCL10^{high}TNFα^{high}Ki67^{+} BV2 in vitro. (**A**) q-PCR detection of *Cxcl10*, *Tnf*, and *Mki67* expression in BV2 with co-stimulation of TNFα (10 ng/mL, the same below), IFNγ (10 ng/mL, the same below), and pRBC (pRBC: microglia = 20: 1, the same below) for 12 h. Data are expressed as mean ± SD; unpaired t-test; $n = 3$.

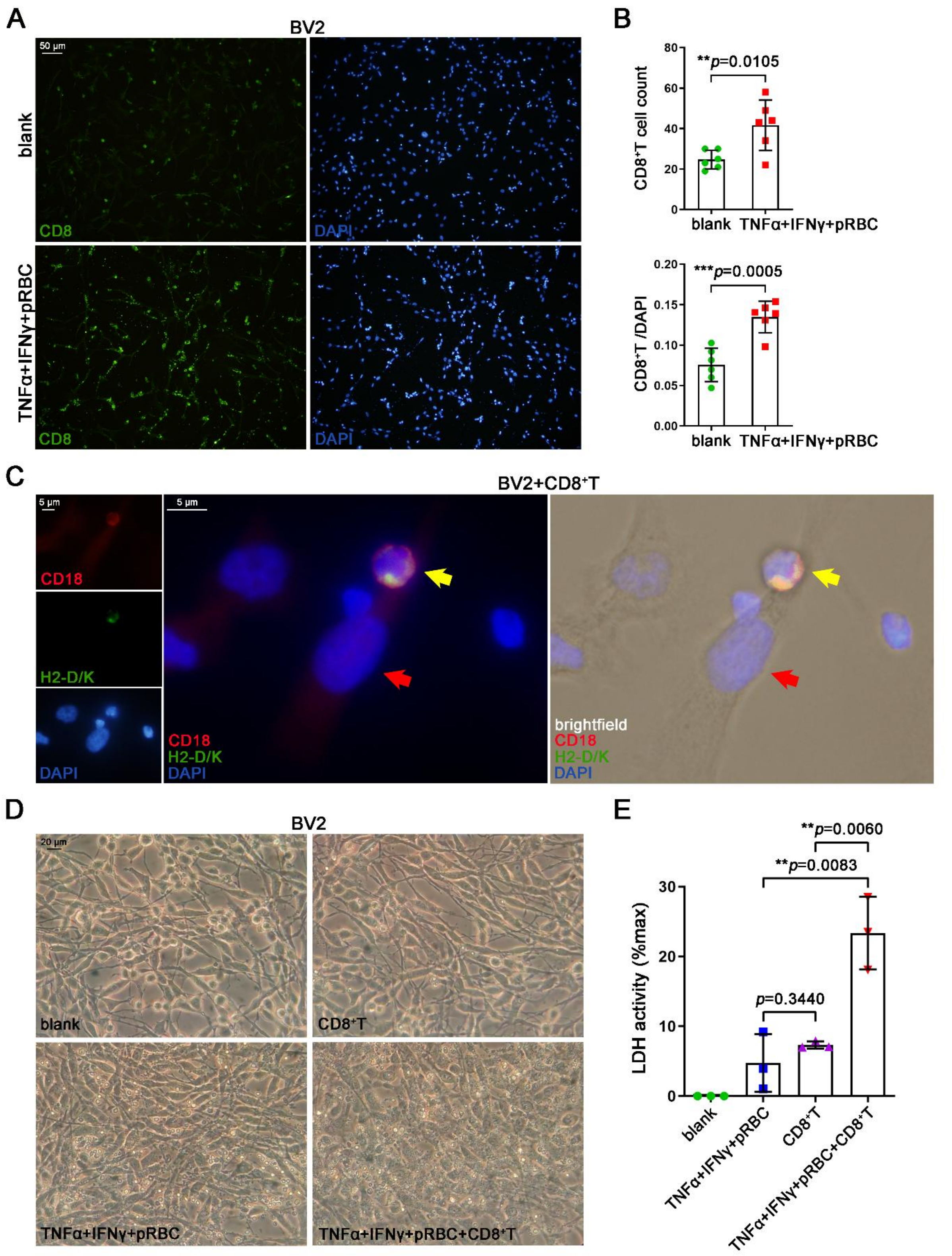

Fig. S4 Interaction between CXCL10^{high}TNFα^{high}Ki67^{+} BV2 and CD8^{+} T cells in vitro. (**A**) IF staining of CD8^{+} T cells recruited in the lower chamber of Transwell system, with CXCL10^{high}TNFα^{high}Ki67^{+} BV2 cultured in the lower chamber. (**B**) Statistical graph of recruited CD8^{+} T cell count in supplementary Fig. 4A. Data are expressed as mean ± SD;

unpaired t-test; n = 6 fields per group. (**C**) IF staining of CD18 and H2-D/K after co-culture of CD8$^+$ T cells (yellow arrow) and CXCL10^{high} TNFα^{high} Ki67$^+$ BV2 (red arrow) for 4 h. (**D**) Optical microscope observation of the CXCL10^{high}TNFα^{high}Ki67$^+$ BV2 co-cultured with CD8$^+$ T cells for 24 h. (**E**) LDH relative activity in supernatants of CXCL10^{high}TNFα^{high}Ki67$^+$ BV2 co-cultured with CD8$^+$ T cells for 24 h. Data are expressed as mean ± SD; unpaired t-test; n = 3.

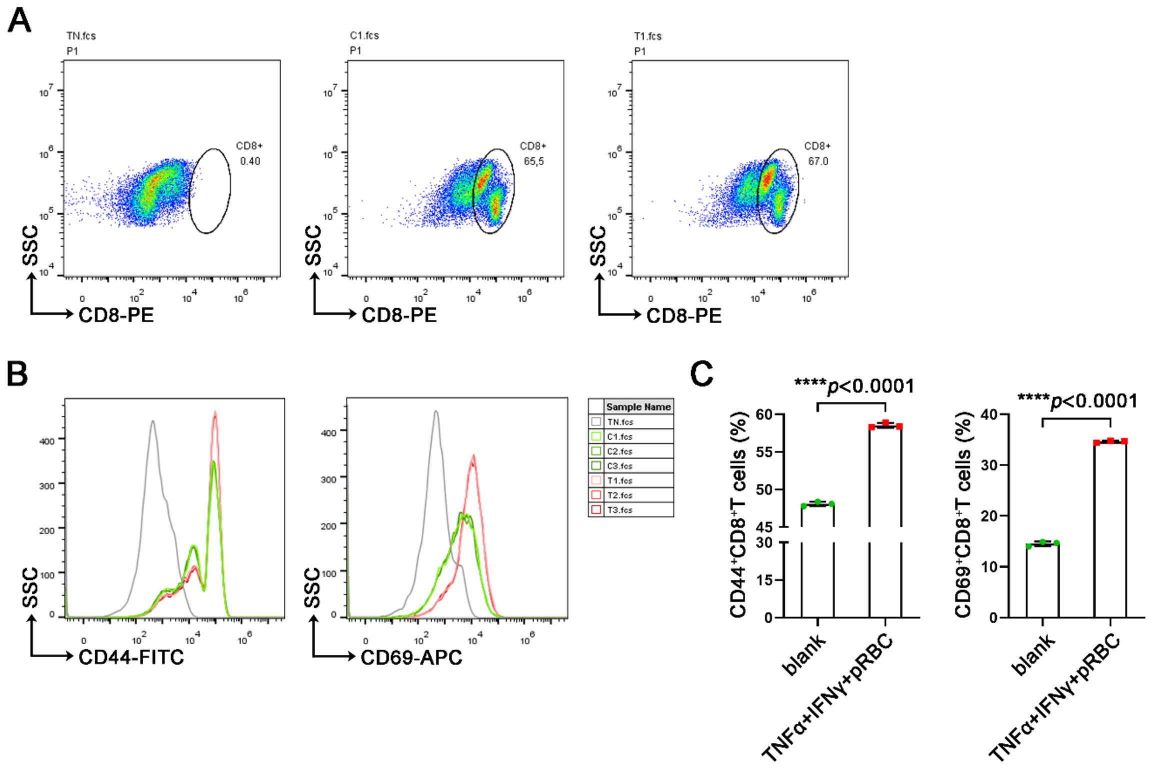

Fig. S5 Sustained activation of CD8$^+$ T cells in vitro detected by flow cytometry. (**A**) CD8$^+$ T cell populations classified using FC. (**B**) FC detection of CD44$^+$CD8$^+$ T cells and CD69$^+$CD8$^+$ T cells after co-culture with CXCL10^{high} TNFα^{high} Ki67$^+$ microglia for 24 h. (**C**) Statistical graph of the proportion of CD44$^+$ CD8$^+$ T cells and CD69$^+$CD8$^+$ T cells in CD8$^+$ T cells in Supplementary fig. 5B. Data are expressed as mean ± SD; unpaired t-test; n = 3.

Table S1 Primers used for q-PCR in this study

Gene	Forward primer	Reverse primer
Cxcl10	CCAAGTGCTGCCGTCATTTTC	GGCTCGCAGGGATGATTTCAA
Tnf	GACGTGGAACTGGCAGAAGAG	TTGGTGGTTTGTGAGTGTGAG
Mki67	AGCACAAAGAGACGGTCTAAGA	CTCTGCCTCGTGACTGTGTT
β-actin	CATCCGTAAAGACCTCTATGCCAAC	ATGGAGCCACCGATCCACA
